# The mossy north: an inverse latitudinal diversity gradient in European bryophytes

**DOI:** 10.1038/srep25546

**Published:** 2016-05-06

**Authors:** Rubén G. Mateo, Olivier Broennimann, Signe Normand, Blaise Petitpierre, Miguel B. Araújo, Jens-C. Svenning, Andrés Baselga, Federico Fernández-González, Virgilio Gómez-Rubio, Jesús Muñoz, Guillermo M. Suarez, Miska Luoto, Antoine Guisan, Alain Vanderpoorten

**Affiliations:** 1 Department of Ecology and Evolution, University of Lausanne, Biophore, CH-1015, Lausanne, Switzerland; 2 Institute of Botany, University of Liège, B-4000, Liège, Belgium; 3 Institute of Environmental Sciences, University of Castilla-La Mancha, ES-45071, Toledo, Spain; 4 Section for Ecoinformatics & Biodiversity, Department of Bioscience, Aarhus University, Ny Munkegade 114, DK-8000, Aarhus C, Denmark; 5 Department of Biogeography and Global Change, National Museum of Natural Sciences (CSIC), ES-28006, Madrid, Spain; 6 InBIO/CIBIO, University of Évora, Largo dos Colegiais, 7000, Évora, Portugal; 7 Center for Macroecology, Evolution and Climate, Natural History Museum of Denmark, University of Copenhagen, Universitetsparken 15, DK-2100, Copenhagen, Denmark; 8 Department of Zoology and Physical Anthropology, University of Santiago de Compostela, ES-15782, Santiago de Compostela, Spain; 9 Department of Mathematics, University of Castilla-La Mancha, ES-02071, Albacete, Spain; 10 Real Jardí n Botá nico (CSIC), ES-28014, Madrid, Spain; 11 CONICET, Facultad de Ciencias Naturales e I.M.L, UNT, Universidad Nacional de Tucamán, 4000, Tucumán, Argentina; 12 Department of Geosciences and Geography, University of Helsinki, 00014, Helsinki, Finland

## Abstract

It remains hotly debated whether latitudinal diversity gradients are common across taxonomic groups and whether a single mechanism can explain such gradients. Investigating species richness (SR) patterns of European land plants, we determine whether SR increases with decreasing latitude, as predicted by theory, and whether the assembly mechanisms differ among taxonomic groups. SR increases towards the south in spermatophytes, but towards the north in ferns and bryophytes. SR patterns in spermatophytes are consistent with their patterns of beta diversity, with high levels of nestedness and turnover in the north and in the south, respectively, indicating species exclusion towards the north and increased opportunities for speciation in the south. Liverworts exhibit the highest levels of nestedness, suggesting that they represent the most sensitive group to the impact of past climate change. Nevertheless, although the extent of liverwort species turnover in the south is substantially and significantly lower than in spermatophytes, liverworts share with the latter a higher nestedness in the north and a higher turn-over in the south, in contrast to mosses and ferns. The extent to which the similarity in the patterns displayed by spermatophytes and liverworts reflects a similar assembly mechanism remains, however, to be demonstrated.

The existence of a latitudinal diversity gradient (LDG) peaking near the equator and decreasing towards the poles has been a persistent feature during the history of life on earth^1^ (but see ref. 2). This gradient has been quoted as one of the few laws in ecology^3^, and it demonstrates remarkable consistency across geographic areas, scales, habitats, and taxonomic groups^4^,^5^,^6^,^7^. Mounting evidence suggests that this convergence of distribution patterns across taxonomic groups is due to environmental forcing^8^.

On the one hand, macroclimate (primarily energy and water) is postulated to control species richness through the ecological sorting of regional and global species pools according to species climatic tolerances or by affecting rates of speciation^9^. In particular, dry or cold environments are specifically challenging for plants because adaptations must evolve to enable the tolerance or avoidance of extremely low water potentials^10^. In lineages that have successfully adapted to high-latitude environments, increased seasonal variability at higher latitudes is assumed to have resulted in broader thermal tolerances and consequently larger ranges: i.e., Rapoport’s rule^11^.

In addition, habitat heterogeneity promotes species richness because diverse habitats allow greater niche separation and therefore promote species coexistence^12^,^13^,^14^. Habitat heterogeneity, when represented by topographic heterogeneity, is also related to historical factors because mountains facilitate the long-term survival of species by allowing climate tracking via short-distance altitudinal migration^15^.

Investigating the factors determining the distribution of 1016 European plant species, Normand *et al.*^16^ confirmed the key role of extant climate and also demonstrated the crucial role of historical factors. Decreased extinction and increased speciation in climatically stable areas is expected to contribute to shaping extant distribution patterns^17^,^18^. For example, the increase in dung beetle species richness towards lower latitudes in Europe results from both the orderly exclusion of species towards the north, resulting in a higher nestedness of northern assemblages compared with southern assemblages and from the high species turnover caused by steep ecological gradients in southern areas^19^. In addition to climate stability, the time required for colonisation can also contribute to the observed latitudinal species richness gradient^13^,^15^. In particular, latitudinal species richness gradients might result from the incomplete post-glacial recolonisation of high-latitude regions. In this context, less mobile organisms are expected to exhibit steeper latitudinal species richness gradients than vagile organisms^20^.

There are, however, some notable exceptions to these patterns, as groups originating during warmer periods of earth’s history display a steeper latitudinal gradient, whereas groups originating during colder periods display a shallower diversity gradient due to a weak affinity or no affinity for lower latitudes^21^. For example, grasses are among the relatively few higher-order lineages that exhibit a shallow, atypical latitudinal gradient due to the climatic specialisation of particular lineages to cold and arid environments^22^. Similarly, although approximately 50% of extant gymnosperm species occur primarily between the tropics, the diversity of gymnosperms decreases at equatorial latitudes^23^.

A latitudinal species richness gradient was observed in spermatophytes^24^ and in ferns^25^ wherein, however, regional species richness patterns do not always correlate with latitude^26^,^27^, and even challenged in bryophytes, a group with approximately 20,000 species of mosses, liverworts, and hornworts, which represents the second most speciose lineage of land plants after the angiosperms. Similar levels of bryophyte species richness have been repeatedly reported from tropical and extra-tropical areas^28^,^29^,^30^,^31^, and Rozzi *et al.*^32^ even documented an inverted bryophyte species richness gradient that increased towards the pole in southern south America. Such weak, if not inverted, gradients of species richness towards high latitudes appear consistent with three major features of bryophyte biology. First, bryophytes typically fail to radiate in contrasted environments^33^, reducing their opportunities to diversify along the steep ecological gradients found at low latitudes. Second, bryophyte species, including tropical species, are inherently better adapted to cold conditions than are angiosperms^34^. They are universally able to grow at low temperatures, showing a growth reduction of less than 50% at 5 °C compared with growth at their optimal temperatures^35^. Simultaneously, because they are poikilohydric, they are much less well equipped to face drought and warm conditions. Bryophytes therefore exhibit lower temperature optima than higher plants^35^; all temperate and boreal species investigated by Furness & Grime^35^ died when kept continuously at 35 °C, and most shoots died at > 30 °C. Third, bryophyte species display larger geographic ranges than angiosperm species and display a high dispersal capacity, resulting in a much lower global rate of species turn-over than angiosperms^36^, suggesting that rapid post-glacial recolonisation prevents the formation of the richness gradients that result from limited post-glacial dispersal processes, as has been observed in vascular plants^15^.

Here, we examined the spatial variation in species richness and beta-diversity (disentangling its two components: species replacement or turnover, and species loss or nestedness) in European bryophytes and compared them with those observed in ferns and spermatophytes. Specifically, we tested three hypotheses: 1) as a result of differences in temperature optima and drought tolerance between bryophytes and spermatophytes, bryophyte species richness should decrease towards lower latitudes, whereas spermatophytes should display the opposite pattern; 2) given the failure of bryophytes to radiate in ecologically contrasted areas with a long history of climate stability such as the Mediterranean, and given their large range sizes, the spatial turnover of bryophyte richness across latitudinal gradients should be less pronounced than in spermatophytes; 3) in spermatophytes, nestedness should increase in the north as a result of limitations in the ability to evolve adaptations to cold conditions and the consequent ordered loss of species towards the north, whereas species turn-over should increase in the south. In bryophytes, we expect to observe a reverse pattern as a result of the inherent cold tolerance in the north and the filtering-out of species towards the south.

## Results

**Figure 1:**
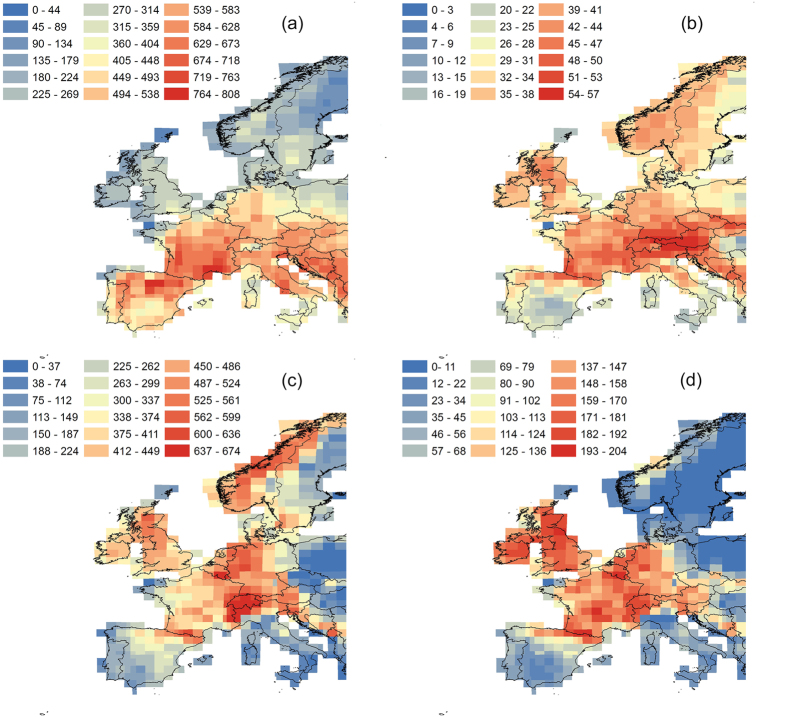
Potential species richness of spermatophytes (a), ferns (b), mosses (c) and liverworts (d) across Europe. Maps based on ensemble stacked species distribution models (S-SDMs) of 1359, 79, 810 and 224 species of spermatophytes, ferns, mosses and liverworts, respectively. Maps generated by R.G. Mateo using the ArcMap extension in ArcGIS 10.2 (ESRI Inc., Redlands, CA, USA, http://www.esri.com).

**Figure 2:**
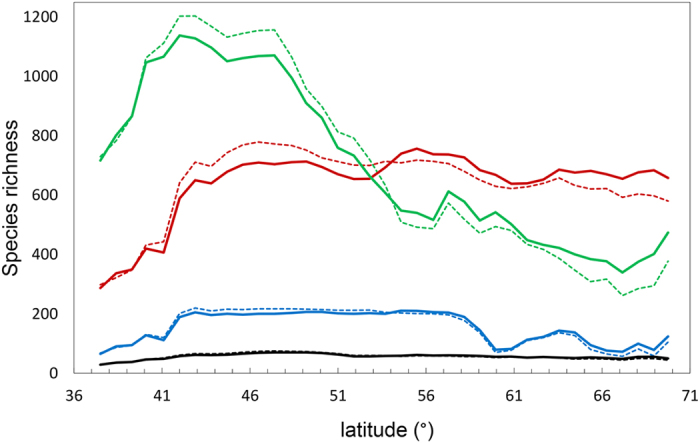
Predicted numbers of species of spermatophytes (green), ferns (black), mosses (red) and liverworts (blue) in 100 km latitudinal bands across Europe. Dashed lines indicate crude SR values predicted by S-SDMs, solid lines correspond to SR values normalized according to species-area relationships (see Supplementary Methods 5).

**Figure 3:**
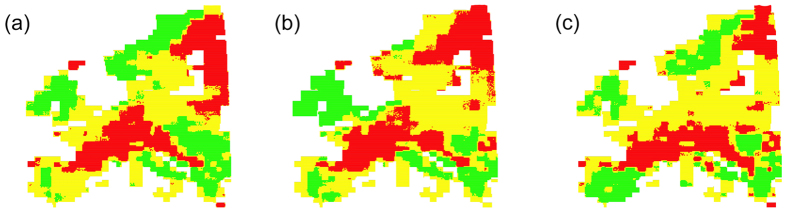
Correlation between the species richness of taxonomical groups across Europe corrected for spatial autocorrelation, as measured by re-scaled Lee’s L bivariate spatial association. Regions of significant spatial association using a Monte Carlo test on Lee’s statistic at the 95% level. ‘Positive’ indicates values of the Lee’s statistic ranked in the top 97'5% of Monte Carlo values, whilst ‘Negative’ indicates a statistic ranked among the bottom 2'5% Monte Carlo values. Maps generated by V. Gómez-Rubio using R 3.2.2 (R Core Team, https://www.r-project.org). (**a**) Correlation between mosses and spermatophytes. (**b**) Correlation between liverworts and spermatophytes. (**c**) Correlation between ferns and spermatophytes.

Consistent with our first hypothesis, European ferns, mosses, liverworts and spermatophytes exhibited contrasting patterns of species richness around a central axis running approximately through the chains of the Pyrenees and the Alps, close to the 46^th^ parallel (Figs 1 and 2). Ferns, mosses and liverworts exhibited a similar impoverishment of species richness towards the Mediterranean and a peak of richness at mid-latitudes that reaches Scandinavia in ferns and mosses, but not in liverworts (Figs 1 and 2). Thus, regions of significant spatial association in the distribution of the four taxonomic groups are distributed along this central axis, whereas negative associations are found southwards and northwards of it (Fig. 3).

**Figure 4:**
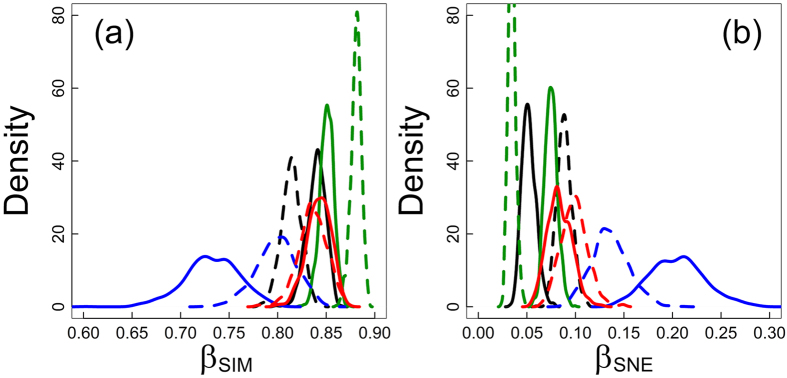
Density plots representing (a) the distribution of the turnover (β _SIM_) and (b) nestedness-resultant multiple-site dissimilarity (β _SNE_) across 1000 samples of 50 pixels. Components of multiple-site dissimilarity were computed for potential species composition in mosses (red), liverworts (blue), ferns (black) and spermatophytes (green) in northern (latitude above 46th parallel, solid line) and southern Europe (latitude below 46th parallel, dashed line).

These differences in species richness patterns among taxonomic groups were paralleled by substantial differences in their patterns of species turnover (β _SIM_) and nestedness-resultant dissimilarity (β _SNE_) in northern (> 46° N) and southern areas (< 46° N) (Fig. 4, Tables 1 and 2). In spermatophytes, β _SIM_ was significantly higher in the south than in the north (*p* <  0.01). Spermatophytes thus exhibited significantly and substantially higher β _SIM_ than ferns, mosses and liverworts in the south (*p* =  0.001), but not in the north. Liverworts exhibited a similar trend as spermatophytes, with β _SIM_ in the south being significantly higher than in the north (*p* =  0.026). Mosses and ferns exhibited the reverse pattern, with significantly higher (*p* =  0.036 in ferns) or similar (*p* =  0.37 in mosses) β _SIM_ in the north than in the south. Thus, there was no difference of β _SIM_ between mosses and ferns in the north (*p* =  0.429) or in the south (*p* =  0.111), whereas both groups exhibited significantly higher β _SIM_ than liverworts in the north (*p* <  0.001 in both mosses and ferns), but not in the south (*p* =  0.067 in mosses and 0.266 in ferns).

In turn, β _SNE_ was significantly higher in the north than in the south (*p* <  0.001) in spermatophytes. Again, although β _SNE_ was significantly higher for ferns, mosses and liverworts than for spermatophytes in the south (*p* <  0.001), whereas the opposite trend was observed in the north (*p* <  0.001 in mosses and liverworts and *p* =  0.011 in ferns), liverworts exhibited a similar trend to spermatophytes as β _SNE_ in the north was significantly higher than in the south (0.019). The opposite trend was observed in mosses and ferns, wherein β _SNE_ was significantly lower in the north than in the south (*p* <  0.001) in the latter, and similar in the north and the south (*p* =  0.220) in the former. Thus, β _SNE_ was significantly higher for liverworts than for mosses and ferns in the north (*p* <  0.001).

## Discussion

Although some previous studies report shallow increases in species richness towards low latitudes^21^ and even of inverted patterns in specific taxonomic groups^37^, we report here a marked increase in species richness towards high latitudes for an entire phylum of land plants at the continental scale. The investigated area in the present study did not encompass the tropics, so that the hypothesis of a global LDG in bryophytes cannot be rejected (but see ref. 29). Nonetheless, our results contrast with the suggestion of a consistent LDG pattern across geographic areas, scales, habitats and taxonomic groups^4^,^5^,^6^,^7^. This pattern was revealed by the analysis of both raw data and stacked species distribution models (see Methods), reducing the potential effect of dispersal limitations on extant species richness patterns. Thus, in contrast with less-mobile organisms, for which historical factors can account for an inverted LDG^37^, major ecological factors also contribute to the observed patterns of increased species richness towards high latitudes in European bryophytes, consistent with the suggestion that habitat suitability and diversity prevail over historical factors (time, speciation, dispersal) in explaining patterns of biodiversity for bryophytes^38^.

In spermatophytes, the marked increase in SR towards the south is paralleled by an increase in species turnover and a reduced nestedness compared with northern areas. The higher spatial species turnover in southern vs. northern areas of Europe is consistent with the strong topographical variation in the Mediterranean basin and with the long-term isolation of specialised populations along ecological gradients, which have accumulated mutations within a relatively stable environment resulting in high rates of local endemism^39^. The markedly lower levels of species turnover in southern mosses, liverworts and ferns compared with southern vascular plants is consistent with our second hypothesis that, unlike spermatophytes, ferns and bryophytes have failed to radiate *in situ* along the strong ecological gradients of the Mediterranean. The significantly higher species turnover observed in southern liverworts as compared to northern ones points, however, to the large difference between assemblages dominated by leafy species in the most humid areas as compared to the assemblages dominated by highly specialized thalloid species such as *Riccia*, characterized by an annual life-cycle and very large spores able to persist underground during the drought season in the most xeric areas^40^.

The higher levels of nestedness-resultant dissimilarity observed in northern spermatophytes compared with southern ones point, in turn, to the exclusion of species from northern areas, in support of our third hypothesis that the failure to evolve adaptations to cold climates is a key mechanism of the LDG in this group^22^. Ferns and mosses exhibited the reverse trend. Fern assemblages, whose distribution and richness patterns are indeed mainly controlled by precipitation levels^41^,^42^,^43^, were significantly more dissimilar due to nested patterns in the south than in the north, pointing to the exclusion of drought-intolerant species from the south. Moss assemblages, however, did not exhibit significantly higher levels of nestedness-resultant dissimilarity in the south than in the north. Since beta diversity, which represents the slope in species-area relationships^44^, does not significantly vary across latitudes in mosses, low levels of moss species richness in the south must be interpreted in terms of the lower carrying capacity (i.e., the intercept of the species-area relationship) of southern areas due to the severe constraint of the poikilohydric condition.

The significant difference in beta diversity along a latitudinal gradient among land plants sheds light on the question of whether the turn-over in community composition is progressively slower from spermatophytes, ferns, and bryophytes, in relationship with the difference in dispersal capacities between these groups^36^, and suggests different mechanisms of assembly in these groups. Liverworts, in particular, strikingly differed from all groups by exhibiting the highest levels of nestedness both in the north and in the south. Such a pattern suggests that liverworts are the most sensitive group to the impact of past climate change, and in particular, to the higher levels of drought that characterized the glacial periods of the Quaternary and particularly affected frost- and drought-sensitive taxa^45^. Nevertheless, although the extent of species turnover in the south was substantially and significantly lower than in spermatophytes, liverworts exhibit a similar pattern towards higher species turnover in the south than in the north, strikingly differing from mosses and ferns in this respect. The patterns of beta diversity displayed by ferns and mosses as compared to liverworts observed here is reminiscent of the differences in the slope of the species-area relationships in liverworts as compared to mosses and ferns^36^. In turn, the similarity in the patterns of beta diversity between liverworts and spermatophytes is puzzling. The extent to which the similarity in the patterns displayed by spermatophytes and liverworts along the latitudinal gradient reflects a similar mechanism of assembly remains, however, to be demonstrated. In the mid-western islands of the Canaries, Madeira and Azores for example, congeneric endemic species generally result from the diversification of a single common ancestor in angiosperms (cladogenetic speciation), but from several independent colonization events in bryophytes (anagenetic speciation)^46^. This suggests that, in the Mediterranean, the diversity of genera such as *Riccia* may not necessarily result, like in angiosperms, from a local radiation, but from the recurrent recruitment of pre-adapted species from south-west Asia, where these genera are highly diversified^47^. While the evolutionary processes underlying the unique diversity of Mediterranean angiosperms have been thoroughly studied^39^, the evolutionary history of the highly specialized Mediterranean liverwort flora remains a large avenue of research to better understand how poikilohydric organisms may thrive and diversify in dry environments.

## Methods

### Implementation of species distribution models to circumvent sampling bias

Although Europe is arguably the continent for which the information on species distributions is most detailed, the corresponding databases and richness patterns are, even in the best-known groups such as vascular plants, globally biased because some areas have been more intensively investigated than others^48^. This problem is exacerbated in less-studied organisms such as bryophytes^49^. To circumvent this issue, we employed species distribution models (SDMs)^50^, which have become a powerful tool for generating maps of potential distribution, or ecological suitability, in areas where distribution information is scarce or lacking.

Available bryophyte distributions include 113,321 records for 1726 species (see Supplementary Methods 1). After removal of the species with less than 15 presences, the data contained 1040 species (including 224 of the 453 liverwort (49%), 810 of the 1,292 moss (63%) and 6 of the 8 hornwort species (73%) of Europe) at a 100 km pixel resolution. Bryophyte species richness was split into mosses and liverworts, two lineages of about 12,000 and 5,000 species. Hornworts should, for consistency, have also been analysed separately. Hornworts are, however, a small group of only about 250 species worldwide whose diversity pales in comparison to the much more diverse liverworts and mosses. The number of hornwort species in our data set did not warrant separate analyses and, because hornworts exhibit a suite of functional vegetative traits and ecological features that are similar to those of thalloid liverworts, the data from the two groups were merged (hereafter referred to as liverworts). Species that were present in fewer than 15 pixels were removed, leaving a total of 1040 species of bryophytes, representing 58% of the total number of species in Europe.

We ran an ensemble model using three different techniques: Generalised Linear Models, Maxent, and Random Forests, as implemented in the R (R Core Team^60^) package BIOMOD 2.0^51^ (see Supplementary Methods 1). We used the 35 macroclimatic variables of CliMond^52^ as environmental predictors, as well as monthly and annual potential evapotranspiration^53^. To avoid multicollinearity, we ran a Pearson correlation analysis eliminating one of the variables in each pair with a correlation value greater than 0.8, as advised by Dormann *et al.*^54^. A final set of six variables was used to run the models (see Supplementary Methods 1). For proper evaluation, the models were trained on 70% of the data and evaluated on the remaining 30%. This split-sampling was replicated 10 times. For each species, the potential distribution was considered as a consensus across statistical techniques, evaluation indices and thresholds used to binarise continuous predictions. These individual potential species distributions were stacked (S-SDMs, stacked species distributions models^55^) to depict the potential SR across Europe (see Supplementary Methods 1).

To validate the resulting potential SR (see Supplementary Methods 2), we compared the maps generated by S-SDMs with 1) the observed bryophyte richness values from a literature review (Supplementary Methods 3), 2) macroecological models (MEMs) of SR for the same study area^56^ and 3) a sampling effort map for bryophytes in Europe (see Supplementary Methods 3).

To obtain comparable results for bryophytes and vascular plants, we further generated a potential richness S-SDM for ferns and spermatophytes (see Supplementary Methods 1). Data for 2,728 native spermatophyte and fern species from the Atlas Florae Europaeae at 50 km pixel resolution^16^ were upscaled to a 100 km pixel resolution for consistency with the bryophyte data. Gymnosperms should have been analysed separately but, as in the case of hornworts (see above), the low number of species (20) did not warrant a specific analysis, so that spermatophytes were analysed globally. After removal of the species with less than 15 presences, the data included 1,359 and 79 spermatophyte and fern species, representing 12% and 49% of their total diversity in Europe, respectively. The potential richness generated by the S-SDM was then compared with the observed richness values for all the species available in this study (see Supplementary Methods 2)^57^.

The predictions of the S-SDMs were highly correlated with the observed richness values and the potential richness values of the macroecological models (MEMs, Supplementary Methods 2), supporting the notion that S-SDMs appear as a very promising tool for modelling species assemblages and providing reliable predictions of the geographical variation in species richness^55^,^58^. Moreover, these predictions showed only a low correlation with the map of sampling effort, indicating that the effects of sampling bias were adequately removed (Supplementary Methods 2).

### Comparison of SR patterns between bryophytes and vascular plants

The potential richness predicted by S-SDMs was employed to compare the spatial patterns of SR between bryophytes and vascular plants, using three approaches: 1) comparison of potential richness maps, 2) spatial turnover and nestedness, and 3) a latitudinal band analysis of SR.

### Comparison of potential richness maps of bryophytes and vascular plants

We began with a comparison of potential richness maps using two different techniques. First, we calculated and mapped the local Lee’s *L* bivariate spatial association^59^ using our own implementation of this statistic with the R language^60^, which is now included in the ‘spdep’ package (Supplementary Methods 4). In contrast to bivariate association measures such as Pearson’s correlation, Lee’s L captures spatial associations among observations in terms of their point-to-point relationships across two spatial patterns.

### Spatial turnover and nestedness in species composition

In the second approach, assemblage multiple-site dissimilarity was measured using the Sørensen index (β _SOR_) and was partitioned into its turnover (β _SIM_) and nestedness-resultant (β _SNE_) components to distinguish between the contribution of spatial species replacement and species loss, respectively^61^, along the environmental gradients. Potential values for β _SIM_ and β _SNE_ were computed independently for mosses, liverworts, ferns, and spermatophytes in northern and southern Europe (defined by the limit of the 46^th^ parallel). Multiple-site dissimilarity was computed 1000 times for randomly sampled subsets of 50 pixels (command beta.sample in R package betapart^62^), and the resulting distributions of β _SIM_ and β _SNE_ values across the 1000 samples were used to empirically assess whether there were significant differences between northern and southern Europe and between mosses, liverworts, ferns and spermatophytes.

### Latitudinal band analysis of species richness

Lastly, we plotted a set of the environmental variables indicated in Table 3 and the potential species richness values for mosses, liverworts, ferns, and spermatophytes for each 100 km latitudinal band across Europe (Supplementary Methods 5).

## Additional Information

**How to cite this article**: Mateo, R. G. *et al.* The mossy north: an inverse latitudinal diversity gradient in European bryophytes. *Sci. Rep.* **6**, 25546; doi: 10.1038/srep25546 (2016).

## Supplementary Material

Supplementary Information

## Figures and Tables

**Table 1:** Species turnover (β_SIM_) and nestedness (β_SNE_) in European spermatophytes (Sp), ferns (Fe), mosses (Mo) and liverworts (Li): significance level (p-value) of the difference between northern (N, latitude above 46th parallel) and southern Europe (S, latitude below 46th parallel) within taxonomic groups (Fig. 4).

	β_SIM_	β_SNE_
Spermatophytes	N < S	N > S
	p < 0.001	p = 0.001
Ferns	N > S	N < S
	p = 0.036	p = 0.001
Mosses	S= N	S = N
	p = 0.370	p = 0.220
Liverworts	N < S	N > S
	p = 0.026	p = 0.019

**Table 2:** Species turnover (β_SIM_) and nestedness (β_SNE_) in European spermatophytes (Sp), ferns (Fe), mosses (Mo) and liverworts (Li): significance level (p-value) of the difference between groups within northern (N, latitude above 46th parallel) and southern Europe (S, latitude below 46th parallel). (Fig. 4).

Taxonomic groups	north		south	
	β_SIM_	β_SNE_	β_SIM_	β_SNE_
Mo vs. Li	Mo > Li	Mo < Li	Mo = Li	Mo = Li
	p < 0.001	p < 0.001	p = 0.067	p = 0.052
Mo vs. Fe	Mo = Fe	Mo > Fe	Mo = Fe	Mo = Fe
	p = 0.429	p = 0.009	p = 0.111	p = 0.253
Mo vs. Sp	Mo = Sp	Mo = Sp	Sp > Mo	Mo > Sp
	p = 0.292	p = 0.278	p = 0.002	p < 0.001
Li vs. Fe	Fe > Li	Li > Fe	Fe = Li	Li > Fe
	p < 0.001	p < 0.001	p = 0.266	p = 0.009
Li vs. Sp	Sp > Li	Li > Sp	Sp > Li	Li > Sp
	p < 0.001	p < 0.001	p < 0.001	p < 0.001
Fe vs. Sp	Fe = Sp	Sp > Fe	Sp > Fe	Fe > Sp
	p = 0.178	p = 0.011	p < 0.001	p < 0.001

**Table 3:** Variables used in the canonical analyses and macroecological models.

Environmental variable	Driver
Mean annual temperature	Kinetic energy
Standard deviation of mean annual temperature	Environmental heterogeneity
Mean annual precipitation in each pixel	Water balance
Standard deviation of mean annual precipitation	Environmental heterogeneity
Standard deviation of altitude	Environmental heterogeneity
Mean potential evapotranspiration in each pixel	Potential energy
Standard deviation of potential evapotranspiration	Environmental heterogeneity
Mean distance to the coast (continentality)	Spatial and climatic
Mean distance to refugia^16^	Spatial and historical

Each variable define a possible driver for species richness patterns.
