# Supplementary material for: The mossy north: an inverse latitudinal diversity gradient in European bryophytes
Source: Sci Rep. 2016 May 6;6:25546. doi: 10.1038/srep25546 (PMC4858760; doi:10.1038/srep25546)
Supplement: Supplementary Information [file srep25546-s1.pdf]

The mossy North: an inverse latitudinal diversity gradient in European bryophytes

R.G. Mateo, O. Broennimann, S. Normand, B. Petitpierre, M.B. Araújo, J.-C. Svenning, A. Baselga, F. Fernández-González, V. Gómez-Rubio, J. Muñoz, G.M. Suarez, M. Luoto, A. Guisan and A. Vanderpoorten

E-mail: rubeng.mateo@gmail.com

Table of contents

- Supplementary Methods 1: Implementation of species distribution models to circumvent sampling bias
- Supplementary Methods 2: Validation of stacked species distribution models
- Supplementary Methods 3: List of references employed to build a network of observed richness patterns for European bryophytes
- Supplementary Methods 4: Comparison of potential richness maps
- Supplementary Methods 5: Comparison of the latitudinal patterns of species richness for mosses, liverworts, ferns and spermatophytes

Supplementary Methods 1: Implementation of species distribution models to circumvent sampling bias

Species distribution models (SDMs) have become a powerful tool to predict distributions in areas where presence points are scarce or lacking[1]. Two features of the present study justify, however, the use of SDMs to predict species richness patterns across Europe. First, error rates diminish drastically when SDMs are applied to large areas that are representative of the distribution ranges of individual species. Second, stacked SDMs were shown to provide reliable predictions of spatial variation in species richness[2].

Species occurrences

Bryophyte distributions include 113,321 records for 1,726 species at 100 km pixel resolution (see ref. 3) following the military grid reference system (MGRS), while data for 2,728 native species of vascular plants from Atlas Florae Europapaeae at 50 km pixel size (see ref. 4) were upscaled to 100 km pixel size for consistency with bryophyte data. Species present in less than 15 pixels were removed and were distributed homogeneously across Europe, leaving a total of 1,438 vascular plants species (1,359 spermatophytes and 79 ferns) and 1,040 bryophytes species (810 mosses and 224 liverworts). Bryophyte sampling bias was removed by random sub-sampling of intensively surveyed areas (see ref. 2).

Atlas of Flora Europeae provides a good representation of vascular plant diversity patterns in western Europe vascular plants, but not in eastern Europe[5-6]. Therefore, eastern MGRS pixels were discarded in following analyses.

Environmental predictors

As environmental predictors we used the 35 macroclimatic variables of CliMond (https://www.climond.org/)[7], as well as monthly and annual potential evapotranspiration (http://www.cgiar-csi.org, see ref. 8). To avoid multicollinearity, we run a Pearson correlation analysis eliminating one of the variables in each pair with a correlation value higher than 0.8. The final set of six variables used to run the models were 'mean diurnal temperature range', 'temperature seasonality', 'precipitation seasonality', 'mean moisture index of warmest quarter', 'mean moisture index of wettest quarter' and 'annual potential evapotranspiration'.

Background selection and statistical modelling

For each species, we generated 10 sets of pseudo-absences equalling the number of presences and sampled from pixels not adjacent to reported occurrences, that were later used to produce an ensemble model[7] using three different techniques: generalized linear models (GLM)[10], Maxent[11], and Random Forests (RF)[12], as implemented in the R[13] package BIOMOD 2.0[14]. The performance of the models was assessed by randomly splitting 10 times the data into a 70% dataset to generate the models and a 30% dataset to estimate their predictive accuracy. After elimination of all models with an AUC<0.8, we generated for each species an ensemble model, consisting in a weighted mean of the models predictions, where the contribution of each individual technique was proportional to its predictive accuracy.

Evaluation statistics and binarization of species' potential distribution

Because different ensemble models could generate different models[15], we generated two ensemble models per species, including either (1) only models with an AUC>0.8 (ROC consensus model), or (2) models with a true skill statistic (TSS) > 0.7 (TSS

consensus model). The contribution of each model to the final ensemble model was proportional to their goodness-of-fit statistics.

If stacking of the binary models reduces the over-prediction[2], the selection of an appropriate threshold still can reduce error rates in both individual and ensemble SDMs[16]. To take into account the effects of the selected threshold used to generate binary models on species' over-prediction, we produce three different binary models per ensemble model, in total of six binary models per species. For the ROC consensus model, we generated models: (1) optimizing the ROC statistics; (2) applying a maximum of 5 % of omission error (i.e. percentage of the presence predicted as absences, omission error[17]; and (3) applying a maximum of 10 % of omission error. For the TSS consensus model, we also generated three richness models: (1) optimizing the TSS statistics; (2) applying a maximum of 5 % of omission error; and (3) applying a maximum of 10 % of omission error.

We stacked (i.e., summed) the different binarised SDMs predictions so that we obtained 6 stacked species distribution models (S-SDMs) per species group (bryophytes and vascular plants), which represent the potential species richness for both groups (Figs. S1 and S2). To compare differences between models, we calculated the Pearson correlation coefficients by pairs of S-SDMs (Tables S1 & S2). As correlations were very high, we discarded possible differences between S-SDMs, so in the following analyses we employed only the S-SDMs obtained by optimizing the ROC statistics for all the subsequent analysis.

Over-fit testing for bryophytes

By definition, rare species distributions usually have few observations, and are prone to over-fitting when modelled. To ensure that models were not over-fitted for large set

of predictors, we employed the ‘ensembling of bivariate models' approach presented by Lomba et al.[18]. It involves generating bivariate models (using only two independent variables every time) and averaging them with a weighted ensemble approach. We have run this approach for bryophytes and climatic variables to compare the results obtained and discard over-fitting problems. As in the original S-SDMs we generated 300 models for every species; in order to be comparable we have run 480 bivariate models per species (4 pseudo-absence sets, 4 replicates, 10 groups of pairs of independent variables, and 3 modelling techniques). We generated a consensus (only models with an AUC>0.8) and a binary model per species (optimizing the ROC statistic). The final richness model generated was compared with the original richness model to discard over-fitting. The Pearson correlation coefficient between both approaches was 0.99, hence we consider that over-fitting problems can be disregarded in the modelling approach followed.

Figure S1. Predicted richness models (S-SDMs) generated for bryophytes. For the ROC consensus model, we generated three models: (1) optimizing the ROC statistics (ROC), (2) applying a maximum 5 % of omission error (ROC.COM5), and (3) applying a maximum of 10 % of omission error (ROC.COM10). For the TSS consensus model, we generated also three models: (1) optimizing the TSS statistics (TSS), (2) applying a maximum of 5 % of omission error (TSS.COM5), and (3) applying a maximum of 10 % of omission error (TSS.COM10). Maps generated by R.G. Mateo using the ArcMap extension in ArcGIS 10.2 (ESRI Inc., Redlands, CA, USA, http://www.esri.com).

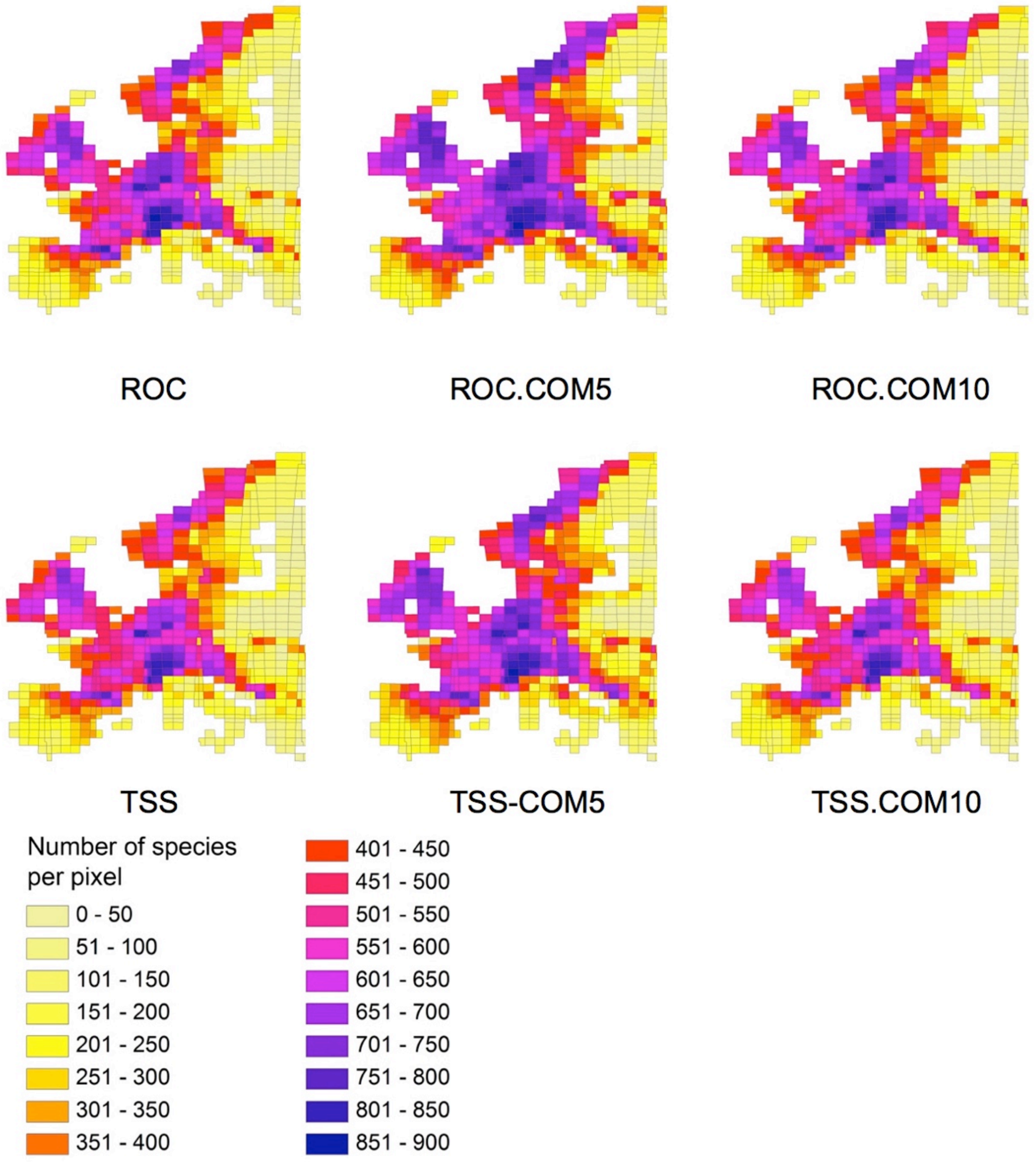

Figure S2. Predicted richness models (S-SDMs) generated for vascular plants. For abbreviations see Figure S1. Maps generated by R.G. Mateo using the ArcMap extension in ArcGIS 10.2 (ESRI Inc., Redlands, CA, USA, http://www.esri.com).

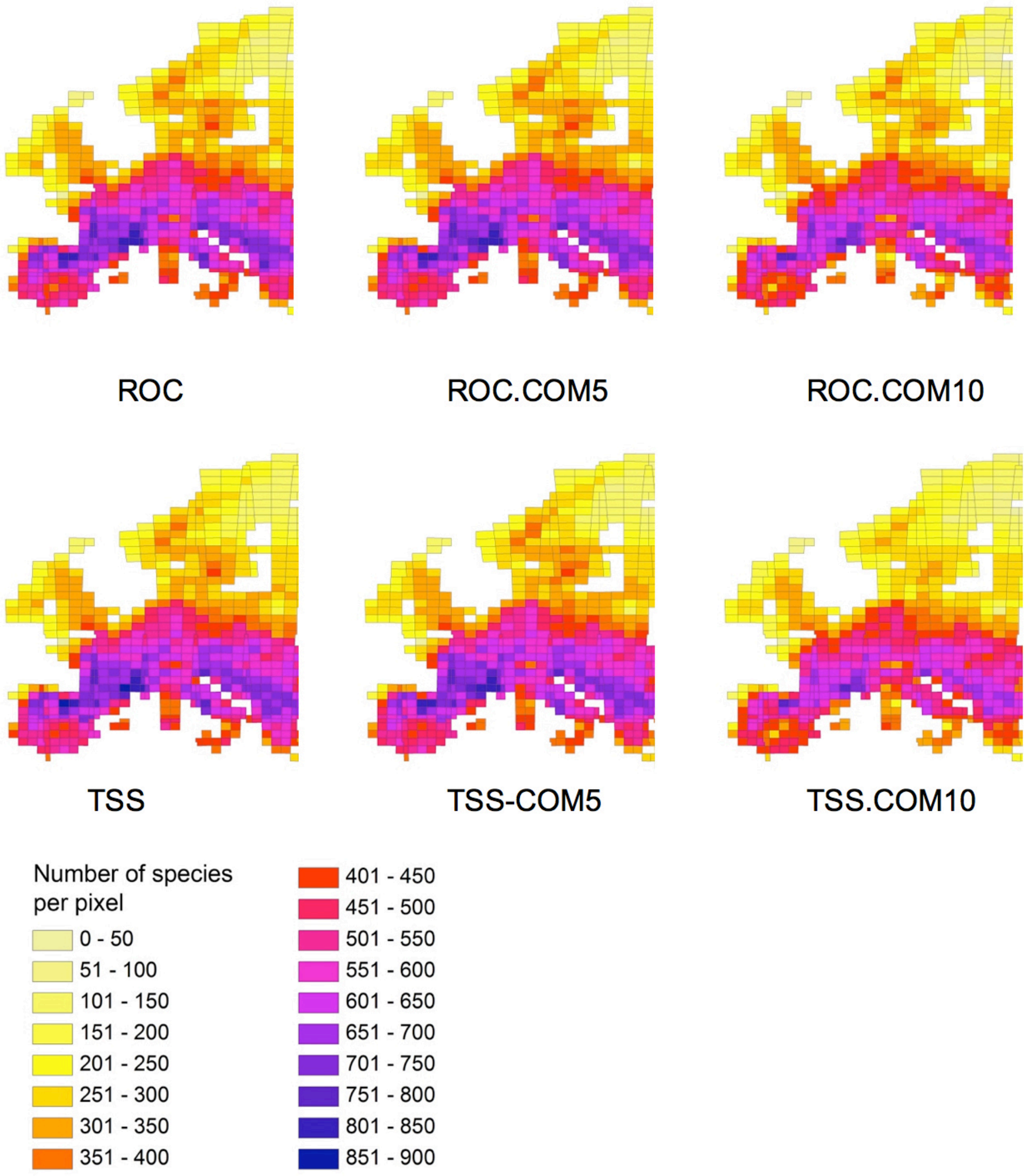

Table S1. Pearson correlation coefficient between all the S-SDMs generated for bryophytes. For abbreviations see Figure S1.

	ROC	ROC.COM5	ROC.COM10	TSS	TSS.COM5	TSS.COM10
**ROC**	1.00	0.98	1.00	1.00	0.99	1.00
**ROC.COM5**	0.98	1.00	0.99	0.98	1.00	0.99
**ROC.COM10**	1.00	0.99	1.00	1.00	1.00	1.00
**TSS**	1.00	0.98	1.00	1.00	0.99	1.00
**TSS.COM5**	0.99	1.00	1.00	0.99	1.00	0.99
**TSS.COM10**	1.00	0.99	1.00	1.00	0.99	1.00

Table S2. Pearson correlation coefficient between all the S-SDMs generated for vascular plants. For abbreviations see Figure S1.

	ROC	ROC.COM5	ROC.COM10	TSS	TSS.COM5	TSS.COM10
**ROC**	1.00	1.00	1.00	1.00	1.00	0.99
**ROC.COM5**	1.00	1.00	0.99	0.99	1.00	0.99
**ROC.COM10**	1.00	0.99	1.00	0.99	0.99	1.00
**TSS**	1.00	0.99	0.99	1.00	1.00	0.99
**TSS.COM5**	1.00	1.00	0.99	1.00	1.00	0.99
**TSS.COM10**	0.99	0.99	1.00	0.99	0.99	1.00

References

1. Guisan, A. & Zimmermann, N.E. Predictive habitat distribution models in ecology. *Ecol. Model.* **135**, 147-186 (2000).

2. Mateo, R. G., Felicísimo, Á. M., Pottier, J., Guisan, A. & Muñoz, J. Do stacked species distribution models reflect altitudinal diversity patterns? *PLoS ONE* **7**, e32586 (2012).

3. Mateo, R. G., Vanderpoorten, A., Muñoz, J., Laenen, B. & Désamoré, A. Modeling species distributions from heterogeneous data for the biogeographic regionalization of the European bryophyte flora. *PLOS ONE* **8**, e55648 (2013).

4. Normand, S. *et al.* Postglaciation migration supplements climate in determining plant species ranges in Europe. *Proc. R. Soc. Lond. B Biol. Sci.* **278**, 3644-3653 (2011).

5. Ronk A, Szava-Kovats R, Pärtel M. Applying the dark diversity concept to plants at the European scale. *Ecography* **38**, 1015–1025 (2015).

6. Kalwij JM, Robertson MP, Ronk A, Zobel M, Pärtel M. Spatially-Explicit Estimation of Geographical Representation in Large-Scale Species Distribution Datasets. *PLoS ONE* **9**, e85306 (2014).

7. Kriticos, D. J. *et al.* CliMond: global high-resolution historical and future scenario climate surfaces for bioclimatic modelling. *Methods Ecol. Evol.* **3**, 53-64 (2011).

8. Zomer, R. J., Trabucco, A., Bossio, D. A. & Verchot, L. V. Climate change mitigation: A spatial analysis of global land suitability for clean development mechanism afforestation and reforestation. *Agric. Ecosyst. Environ.* **126**, 67-80 (2008).

9. Araújo, M. B. & New, M. Ensemble forecasting of species distributions. *Trends Ecol. Evol.* **22**, 42-47 (2007).

10. McCullagh, P. & Nelder, J. A. *Generalized Linear Models*, 2nd ed (Chapman & Hall, London, 1989).

11. Phillips, S. J., Anderson, R. P. & Schapire, R. E. Maximum entropy modeling of species geographic distributions. *Ecol. Modell.* **190**, 231-259 (2006).

12. Breiman, L. Random forests. *Mach. Learn.* **45**, 5-32 (2001).

13. R Core Team (2013). R: A language and environment for statistical computing. R Foundation for Statistical Computing, Vienna, Austria. URL http://www.R-project.org/".

14. Thuiller, W., Lafourcade, B., Engler, R. & Araújo, M. B. BIOMOD - a platform for ensemble forecasting of species distributions. *Ecography* **32**, 369-373 (2009).

15. Marmion, M., Parviainen, M., Luoto, M., Heikkinen, R. K. & Thuiller, W. Evaluation of consensus methods in predictive species distribution modelling. *Divers. Distrib.* **15**, 59-69 (2009).

16. Trotta-Moreu, N. & Lobo, J. M. Deriving the species richness distribution of Geotrupinae (Coleoptera: Scarabaeoidea) in Mexico from the overlap of individual model predictions. *Environ. Entomol.* **39**, 42-49 (2010).

17. Fielding, A. H. & Bell, J. F. A review of methods for the assessment of prediction errors in conservation presence/absence models. *Environ. Conserv.* **24**, 38-49 (1997).

18. Lomba, A. *et al.* Overcoming the rare species modelling paradox: a novel hierarchical framework applied to an Iberian endemic plant. *Biol. Conserv.* **143**, 2647-2657 (2010).

Supplementary Methods 2: Validation of stacked species distribution models

Potential richness maps obtained by stacked species distribution models (S-SDMs) have been rarely evaluated until recently in a few studies[1-3], and never for inconspicuous organism like bryophytes. The first step was to evaluate the S-SDMs obtained to check if the sampling bias was solved. We compared the S-SDMs obtained for bryophytes with i) observed species richness values of bryophytes from a literature review, ii) a macroecological model (MEM)[4], and iii) a sampling effort map for bryophytes in Europe.

For the first comparison we produced a dataset of observed species richness for 45 UTM squares (100x100 km) scattered across Europe and intensively sampled according to a previous review of the literature (Figure S3, see Appendix S5 for the complete list of references).

Then we generated a set of variables describing extant macroclimatic conditions, environmental heterogeneity, spatial patterns and historical factors across Europe (see Table 1 of the main manuscript). We selected the variables that best describe patterns of bryophyte species richness across the 45 UTM squares through a multimodel inference approach[5] approach. We employed the MuMin R package[6] to select the variables with higher relative importance along a set of all the possible generalized linear models in groups of four variables to avoid overparameterization, following the sample size rule-of thumb of 10:1 subjects to predictors in multiple regression[7]. The four variables with higher importance along all the possible GLM models were: continentality, standard deviation of annual temperature, standard deviation of altitude, and distance to refugia. The GLM model run with the four variables obtained the lower AIC value (1851).

The importance of distance to refugia suggests at first sight that species are still concentrated close to the areas least affected by Pleistocene ice age cooling, and hence, exhibit post-glacial dispersal limitations. Such a result was unexpected for two reasons. Fist, the relevance of distance to southern refugia in extant bryophyte species richness patterns implicitly suggests that species survived the ice age in Mediterranean refugia (southern refugium hypothesis) rather than in extra-Mediterranean micro-refugia (northern refugium hypothesis), in contrast to some phylogeographical evidence[8]. Second, such an important contribution of historical factors in extant patterns of bryophyte species richness, also observed in other groups such as angiosperms[9] and reptiles[10], is at odds with the high long-distance dispersal capacities, which were thought to erase any historical signal in extant patterns of species richness[11]. In fact, bryophytes quickly and massively responded to past climate change based on phylogeographic[12, 13] and macrofossil [14, 15] evidence, supporting the view that bryophyte distributions and species richness patterns are at equilibrium with contemporary ecological factors[11]. An important caveat to such interpretations, however, is the strong correlation between variables. For instance, distance to refugia, which appears at first sight as a historical factor, is strongly correlated with evapotranspiration[16], confounding the interpretation that can be made from the selection of the best-fit variables in the model.

The four variables selected were employed as predictors of a macroecological model (MEM)[4] built on the 45 observed species richness values. Generalized linear models (GLM)[17]; R library 'glm') were implemented using a quadratic function, calibrated with a Poisson distribution and a logarithmic link function. This MEM was consequently projected onto Europe to predict bryophyte species richness across the continent (Figure S3).

On the other hand, we downloaded all the collections available for bryophytes in the Global Biodiversity Information Facility (GBIF) database across Europe. We counted the number of collections per UTM squares (100x100 km) to generate a map of sampling effort for bryophytes in Europe (Figure S3).

Finally we calculated the Pearson correlation coefficients between the three maps generated on the 45 pixels with observed richness values from bibliography. Those values were highly correlated with the potential richness obtained by S-SDMs and MEM, and very low correlated with sampling effort. In conclusion, the potential bryophyte richness (S-SDMs) map is not biased by sampling effort, and it is a good tool to study biodiversity patterns for bryophytes in Europe.

We also compare the S-SDMs obtained for vascular plants and observed richness patterns. We generated a map of observed richness patterns (Figure S4) for vascular plants with all the data available in the study[9].

Figure S3: Potential species richness for bryophytes calculated with stacked species distribution models (S-SDMs) and with a macroecological model (MEM), and a map of sampling effort for bryophytes in the GBIF database (number of collections available in the GBIF database for bryophytes in each pixel). Maps generated by R.G. Mateo using the ArcMap extension in ArcGIS 10.2 (ESRI Inc., Redlands, CA, USA, http://www.esri.com).

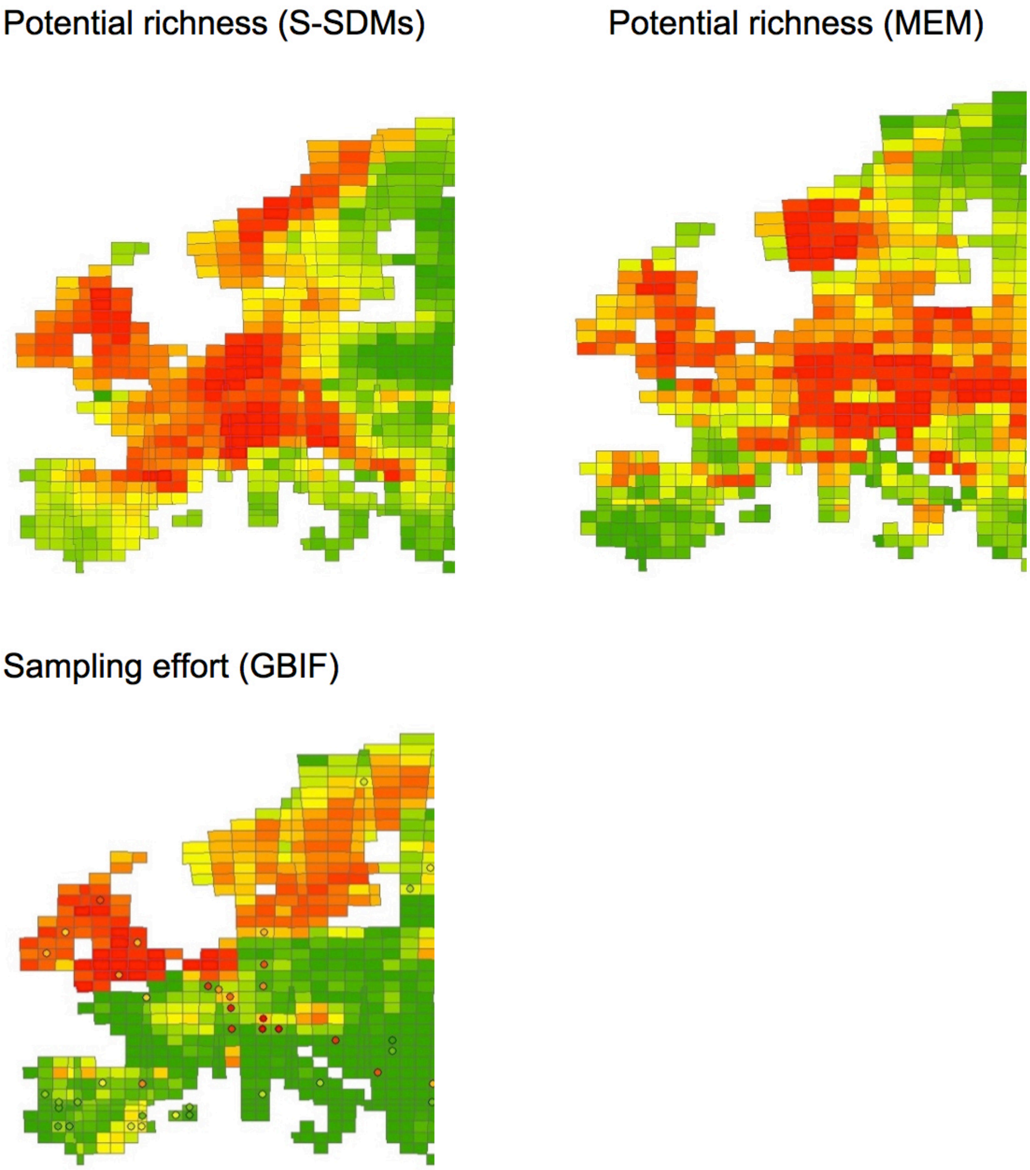

Table S3: Pearson correlation coefficients between observed bryophyte species richness (45 pixels, information extracted from bibliography), potential bryophyte species richness calculated with S-SDMs, potential bryophyte species richness estimated with a MEM, and sampling effort for bryophytes in the GBIF database.

	**Observed**	**S-SDMs**	**GBIF**	**MEM**
**Observed**	1.00	0.91	0.23	0.90
**S-SDMs**	0.91	1.00	0.27	0.88
**GBIF**	0.23	0.27	1.00	0.22
**MEM**	0.90	0.88	0.22	1.00

Figure S4: Potential plant richness obtained by stacked species distribution models (S-SDMs) and observed vascular plant richness in the complete database of Atlas Florae Europaeae (AFE). Maps generated by R.G. Mateo using the ArcMap extension in ArcGIS 10.2 (ESRI Inc., Redlands, CA, USA, http://www.esri.com).

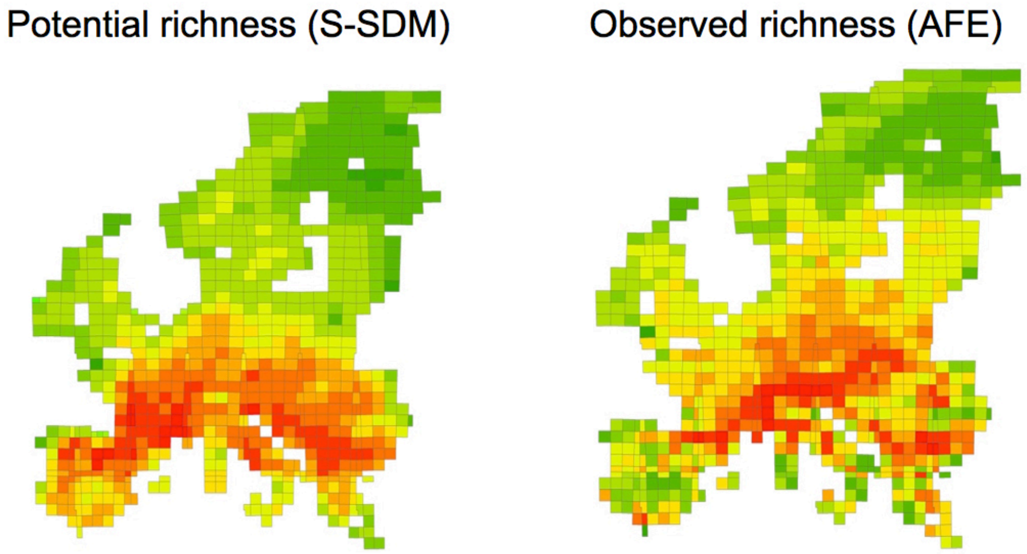

References

1. Dubuis, A. *et al*. Predicting spatial patterns of plant species richness: a comparison of direct macroecological and species stacking approaches. *Divers. Distrib*. **17**, 1122-1131 (2011).

2. Distler, T., Schuetz, J. G., Velásquez-Tibatá, J. & Langham, G. M. Stacked species distribution models and macroecological models provide congruent projections of avian species richness under climate change. *J. Biogeogr*. **42**, 976-988 (2015).

3. Mateo, R. G., Felicísimo, Á. M., Pottier, J., Guisan, A. & Muñoz, J. Do stacked species distribution models reflect altitudinal diversity patterns? *PLoS ONE* **7**, e32586 (2012).

4. Gotelli, N. J. *et al.* Patterns and causes of species richness: a general simulation model for macroecology. *Ecol. Lett*. **12**, 873-886 (2009).

5. Grueber, C. E., Nakagawa, S., Laws, R. J. & Jamieson, I. G. Multimodel inference in ecology and evolution: challenges and solutions. *J. Evol. Biol*. **24**, 699-711 (2011).

6. Barton, K. MuMIn: multi-model inference. R package, version 1.14.0. 2015. URL: http://r-forge.r-project.org/pro-jects/mumin/

7. Harrell, F. E. *Regression Modeling Strategies with Applications to Linear Models, Logistic Regression, and Survival Analysis* (Springer, New York, 2001).

8. Désamoré, A., Laenen, B., Stech, M., Papp, B., Hedenäs, L., Mateo, R.G. & Vanderpoorten, A. How do temperate bryophytes face the challenge of a changing environment? Lessons from the past and predictions for the future. *Glob. Chang. Biol.* **18**. 2915-2924 (2012).

9. Normand, S., Ricklefs, R. E., Skov, F., Bladt, J., Tackenberg, O. & Svenning, J. C. Postglacial migration supplements climate in determining plant species ranges in Europe. *Proc. R. Soc. Lond. B* **278**, 3644-3653 (2011).

10. Araújo, M.B., Nogués-Bravo, D., Diniz-Filho, J.A.F., Haywood, A.M., Valdes, P.J. & Rahbek, C. Quaternary climate changes explain diversity among reptiles and amphibians. *Ecography* **31**. 8-15 (2008).

11. Patiño, J. *et al.* Accounting for data heterogeneity in patterns of biodiversity: an application of linear mixed effect models to the oceanic island biogeography of spore-producing plants. *Ecography*, 36, 904-913 (2013).

12. Van der Velde, M. & Bijlsma, R. Phylogeography of five Polytrichum species within Europe. *Biol. J. Linn. Soc.* **78**. 203-213 (2003).

13. Szövényi, P., Sundberg, S. & Shaw, A.J. Long-distance dispersal and genetic structure of natural populations: an assessment of the inverse isolation hypothesis in peat mosses. *Mol. Ecol.* **21**. 5461-5472 (2012).

14. Jonsgard, B. & Birks, H.H. Late-glacial mosses and environmental reconstructions at Krakenes, western Norway. *Lindbergia* **20**. 64-82 (1996).

15. Ellis, C.J. & Tallis, J.H. Climatic control of blanket mire development at Kentra Moss, north-west Scotland. *J. Ecol.* **88**. 869-889 (2000).

16. Fløjgaard, C., Normand, S., Skov, F. & Svenning, J.-C. Deconstructing the mammal species richness pattern in Europe – towards an understanding of the relative importance of climate, biogeographic history, habitat heterogeneity and humans. *Global Ecol. Biogeogr.* **20** 218-230 (2011).

17. McCullagh, P. & Nelder, J.A. *Generalized linear models* (Chapman & Hall, London, 1989).

Supplementary Methods 3: List of references employed to build a network of observed richness patterns for European bryophytes

1- National Inventory of Swiss Bryophytes of National Inventory of Swiss Bryophytes (Institute of Systematic Botany, University of Zürich): http://www.nism.uzh.ch/map/map_en.php. Available at: (accessed July 2014 2014).

2- Aleffi, M. & Schumacker, R. Check-list and red-list of the liverworts (Marchantiophyta) and hornworts (Anthocerotophyta) of Italy. *Flora Mediterranea*, **5**, 73-161 (1995).

3- Atherton, I., Bosanquet, S. & Lawley, M. *Mosses and Liverworts of Britain and Ireland. A Field Guide*. (British Bryological Society. London, 2010)

4- Cano, J.M., Ros, R.M. & Guerra, J. Flora briofítica de la provincia de Alicante. *Cryptogam. Bryol.* **17**, 251-277 (1996).

5- Cano, J.M., Guerra, J., Jiménez, J.A., Gallego, M.T. & Orgaz, J.D. An updated Bryophytes Check-list of the Region of Murcia (Southeastern Spain). *Anales de Biología* **32**, 101-131 (2010).

6- Cano, M.J., Gallego, M.T., Garilleti, R., Juaristi, R., Lara, F., Abaigar, J.M., Mazimpaka, V., Rosselló, J.A., Sánchez-Moya, M.C. & Urdíroz, A. Aportaciones al conocimiento de la flora briológica española. Notula XIII: hepáticas y musgos de Mallorca (Islas Baleares). *Boletín de la Sociedad Española de Briología* **18/19**, 103-110 (2001).

7- Cogoni, A., Flore, F. & Aleffi, M. Survey of the bryoflora on Monte Limbara (Northern Sardinia). *Cryptogam. Bryol.* **23**, 73-86 (2002).

8- Cortini Pedrotti, C. New Check-list of the Mosses of ltaly. *Flora Mediterranea* **11**, 23-107 (2001).

9- Cros, R.M., Sáez, L. & Brugués, M. The bryophytes of the Balearic Islands: an annotated checklist. *J. Bryol.* **30**, 74–95 (2008).

10- Dragicevic, S., Veljic, M. & Marín, P. New records to the moss flora of Montenegro. *Cryptogam. Bryol.* **29**, 397-400 (2008).

11- Draper, I., Albertos, B., Brugués, M., Cano, M.J., Cros, R.M., Gallego, M.T., Garilleti, R., Guerra, J., Lara, F. & Mazimpaka, V. Aportaciones al conocimiento de la flora briológica española. Notula XIV: musgos, antocerotas y hepáticas de la sierra de Aracena (Huelva). *Boletín de la Sociedad Española de Briología* **24**, 7-14 (2004).

12- Düll, R. *Moosflora der nördlichen Eifel.* (Bad Münstereifel, 1995).

13- Elías, M.J., Albertos, B., Brugués, M., Calabrese, G., Cano, M.J., Estébanez, B., Gallego, M.T., Garilleti, R., Guerra, J., Heras, P., Infantes, M., Lara, F., Martín, M.A., Mazimpaka, V., Medina, R., Muñoz, J., Pokorny, L., Puche, F. & Sánchez, J.A. Aportaciones al conocimiento de la flora briológica española. Notula XV: musgos, antocerotas y hepáticas de la Sierra de Gredos (Ávila). *Boletín de la Sociedad Española de Briología* **28**, 25-31 (2006).

14- Frahm, J.P. *La Bryoflore des Vosges et des zones limitrophes*. Universität-(Gesamthochschule-Duisburg, 1989).

15- Frahm, J.P. & Walsemann, E. Nachträge zur Moosflora von Schleswig-Holstein. Mitt. Arbeitsgem. *Geobotanik Heft* **23**, 1-205 (1973).

16- Ganeva, A. & Natcheva, R. Check-list of the bryophytes of Bulgaria with data on their distribution. I. Hepaticae and Anthocerotae. *Cryptogam. Bryol.* **24**, 229-239 (2003).

17- Heras, P. & Infante, M. Actualización y contribuciones a la brioflora de Burgos. *Boletín de la Sociedad Española de Briología* 27-31 (2004).

18- Ingerpuu, N. & Vellak, K. Bryologically important sites in Estonia. *Lindbergia* **25**, 106 – 111 (2000).

19- Kalinauskaitė, P. & Pippo, S. The bryophyte flora of Bromarv, southwest Finland, based on Hans Buch's Reliquiae and other collections. *Bryobrothera* **9**, 1-49 (2010).

20- Loeske, L. *Mossflora des Harzes*. Bornträger, Leipzig. (1903).

21- Maksimov, A., Potemkin, A., Hokkanen, T. & Maksimova, T. Bryophytes of fragmented old-growth spruce forest stands of the north Karelian Biosphere Reserve and adjacent areas of Finland. *Arctoa* **12**, 9-23 (2003).

22- Mårtensson, O. *Mossflora och mossvegetation krin Sjön Keddek I Taurejuätnodalen I lule Lappmark*. (Stockholm, 1962).

23- Natcheva, R. & Ganeva, A. Check-list of the bryophytes of Bulgaria. II. Musci. *Cryptogam. Bryol.* **26**, 209-232 (2005).

24- Papp, B., Alegro, A., Šegota, V., Šapić, I. & Vukelić, J. (2013) Additions to the bryophyte flora of Croatia. *J. Bryol.* **35**, 140-143.

25- Pérez, P.H., Infante, M., Casas, C., Cros, R.M. & Brugués, M. Contribución a la brioflora del Pirineo Aragonés. *Boletín de la Sociedad Española de Briología* **25**, 25-32 (2004).

26- Puche, F. & Gimeno, C. Flora briofítica de la Muela de Cortes y del Macizo del Caroche (Valencia, España). *Boletín de la Sociedad Española de Briología* **24**, 15–26 (2004).

27- Sabovljević & Cvetić, T. Bryophyte Flora of Avala Mt. (C. Serbia, Yugoslavia). *Lindbergia* **28**, 90-96 (2003).

28- Sabovljevic, M. Bryophyte flora of South Banat. (Vojvodina, Yugoslavia). *Cryptogam. Bryol* **24**, 241-252 (2003).

29- Sabovljevic, M., Tsakiri, E. & Sabovljevic, A. Towards the bryophyte flora of Greece, studies in Chalkidiki area (North Greece). *Cryptogam. Bryol.* **29**, 143-145 (2008).

30- Sérgio, C., Brugués, M., Cros, R.M., Garcia, C. & Louro, T. A new important Mediterranean area for bryophytes in Portugal: Barrancos (Baixo Alentejo). *Boletín de la Sociedad Española de Briología* **29**, 25-33 (2006).

31- Sérgio, C., Brugués, M., Cros, R.M., Garcia, C. & Stow, S. First bryofloristic study of the Tejo International Region (Portugal). *Boletín de la Sociedad Española de Briología* **37**, 1-10 (2011).

32- Vieira, C., Séneca, A. & Sérgio, C. The bryoflora of Valongo. The refuge of common and rare species *Boletín de la Sociedad Española de Briología*, 1-15 (2004).

33- Werner, J. Check-list of the bryophytes of Luxembourg. *J. Bryol.* **17**, 489-500 (1993).

34- Werner, J., Schneider, T., Schneider, C. & Mahévas, T. Les bryophytes de la Lorraine extra-vosgienne. Liste critique annotée. *Cryptogam. Bryol.* **26**, 347-402 (2005).

35- Werner, J., Bardat, J., Vanot, M. & Prey, T. Bryophyte (Anthocerotae, Hepaticae, Musci) check-list of upper Normandy (France). *Cryptogam. Bryol.* **30**, 457-475 (2009).

Supplementary Methods 4: **Comparison of potential richness maps**

Potential richness (stacked species distribution models, S-SDMs) maps were employed to compare the spatial patterns of species richness among mosses, liverworts, ferns and spermatophytes. The first step was two couple pixel by pixel richness patterns analyses.

First, we calculated and mapped local Lee's *L* bivariate spatial association[1] using our own implementation of this statistics with the R language. This function is now included in the 'spdep' package[2]. Lee's L statistics is a measure of the pixel-to-pixel correlation between two spatial variables, corrected for the spatial autocorrelation of each variable. As Moran's I index, Lee's L is not necessarily centered at 0, and its limits can be outside of the (-1,1) interval[3]. To facilitate the interpretation of the L statistics, we centred it on 0 and fixed its boundaries between -1 and 1 by subtracting the mean of the local Lee's *L* values and dividing the result by the maximum value that *L* could take. An associated quantile was computed for each value of local Lee's *L* statistics using a Monte Carlo test with 999 simulations so that significant positive (quantile >0.975) or negative (quantile <0.025) spatial association can be detected.

To complement this analysis, we mapped the residuals of a Procrustes[4] analysis with the "vegan" R package. Procrustes rotation allows the comparison of the ordinations of two data matrices through an algorithm that minimises the sum of squared distances between corresponding points belonging to the two matrices. Procrustean comparison was applied to two canonical correspondence analyses (CCAs)[5] performed with the ade4 R-package[6] and conducted using the matrices of spermatophytes, mosses, liverworts and ferns species probabilities in each 100 km pixel against a set of environmental variables (Table 1) capable of explaining species composition patterns. High residual values indicate areas where the major gradients

differ, i.e., where the influence of the environmental variables differed for spermatophytes and the other three groups.

The CCA with spermatophytes and environmental variables produced two axes, the first one expressing the temperature, and the other one environmental heterogeneity. The highest residual values are given in the most heterogeneous areas (Alps and Pyrenees), so in this area composition is mostly explained with these environmental variables. The CCAs with ferns, mosses or liverworts and environmental variables produced two axes, the first one expressing the temperature, and the other one potential evapotranspiration and environmental heterogeneity. Inertia of spermatophytes, ferns, mosses, and liverworts composition explained by environmental variables was 65.05%, 68.67%, 67.44%, and 66.13%, respectively. The CCA gets the most important gradients of the independent variables to explain the richness patters for each group (Fig. S6), and the Procrustes analysis (Fig. S7) compare both CCA to see if they are the same. High differences between gradients (high values of residuals) indicate that environmental variables have different influence for the taxonomical groups compared.

Figure S5. Correlation between the species richness of mosses, liverworts, ferns and spermatophytes across Europe corrected for spatial autocorrelation, as measured by re-scaled Lee's L bivariate spatial association. Regions of significant spatial association using a Monte Carlo test on Lee's statistic at the 95% level. 'Positive' indicates values of the Lee's statistic ranked in the top 97'5% of Monte Carlo values, whilst 'Negative' indicates a statistic ranked among the bottom 2'5% Monte Carlo values. Maps generated by V. Gómez-Rubio using R 3.2.2 (R Core Team, https://www.r-project.org). (a) Correlation between mosses and spermatophytes. (b) Correlation between liverworts and spermatophytes. (c) Correlation between ferns and spermatophytes.

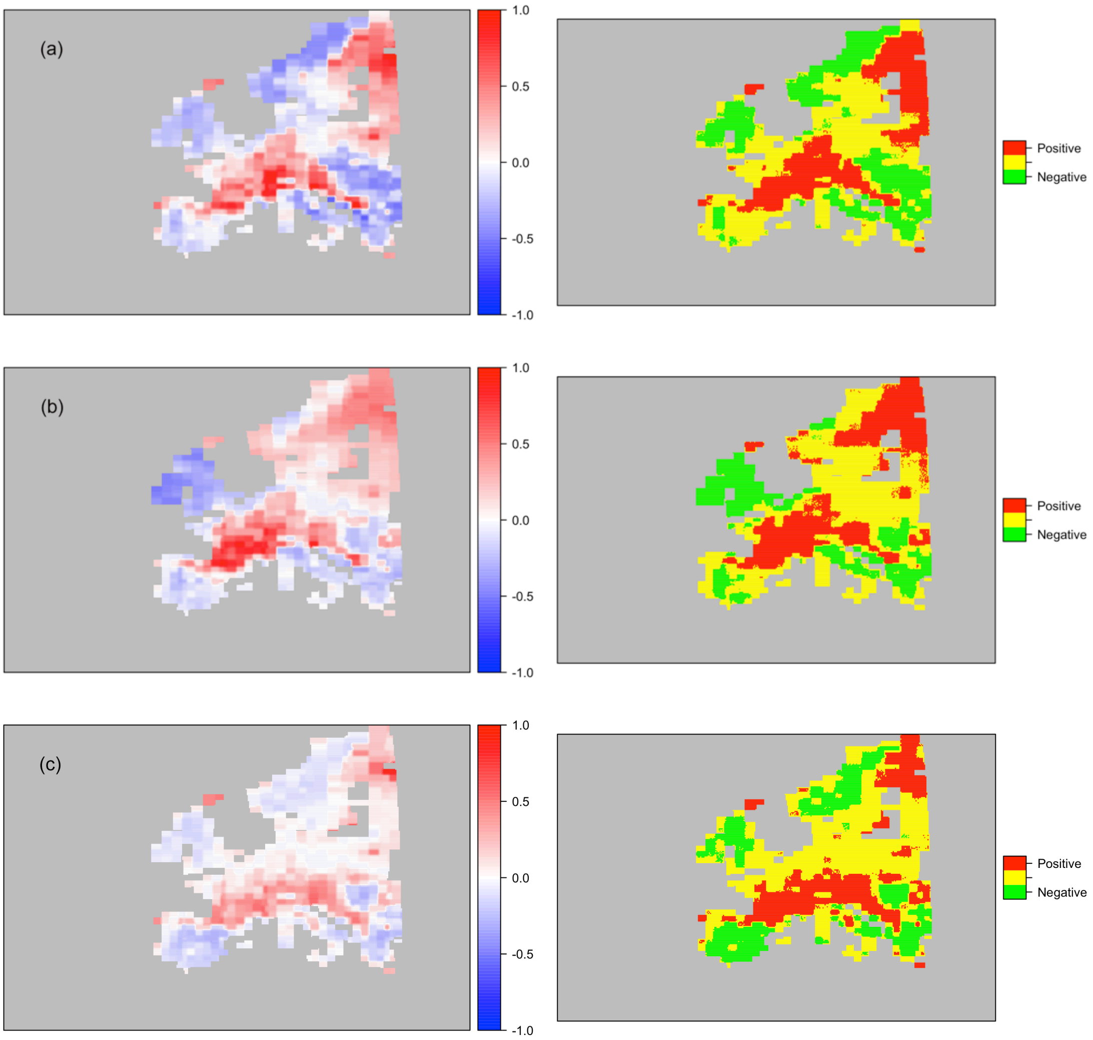

Figure S6a. Results of canonical correspondence analysis of species composition of spermatophytes and environmental variables (see Table 1).

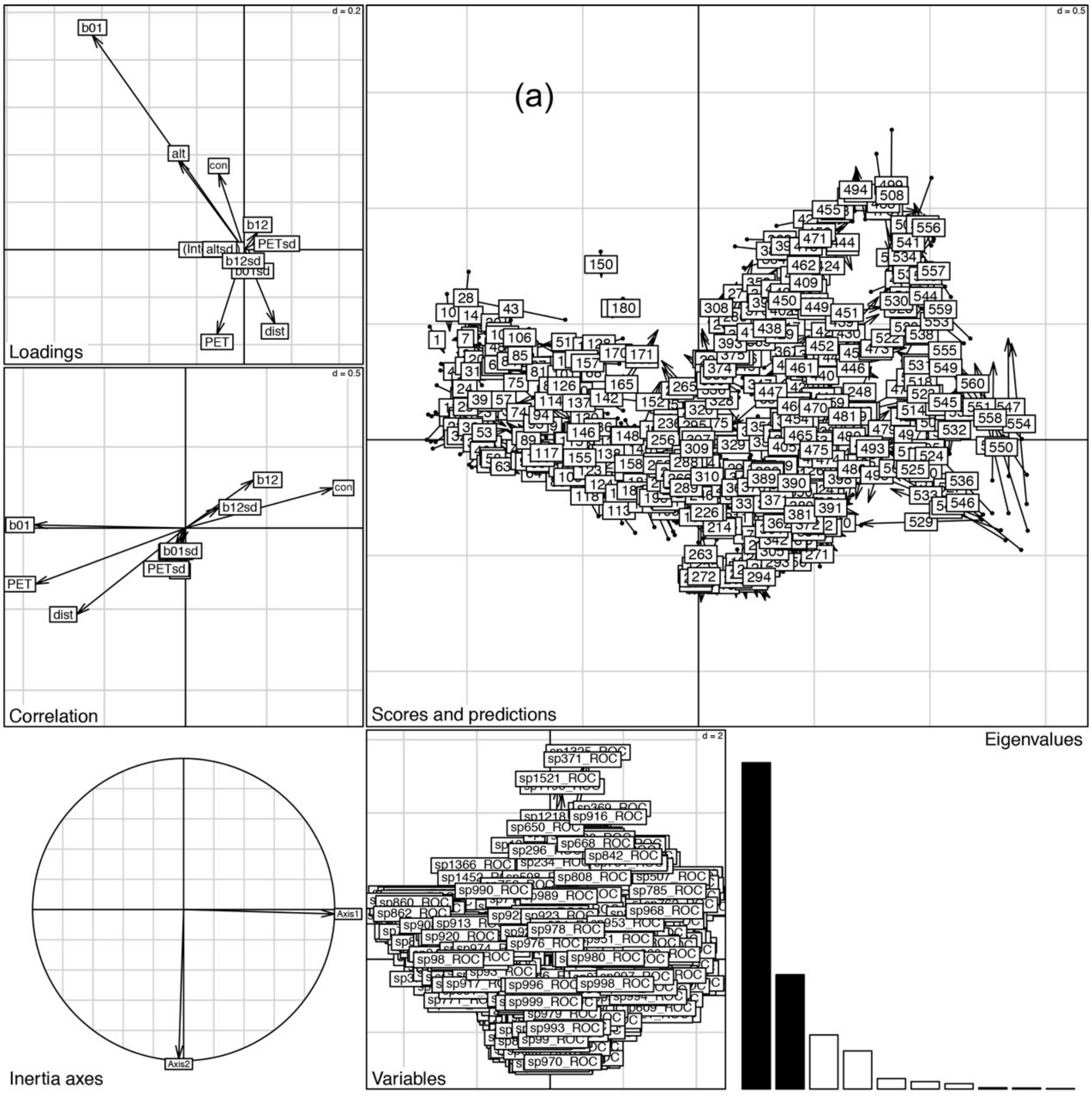

Figure S6b. Results of canonical correspondence analysis of species composition of ferns and environmental variables (see Table 1).

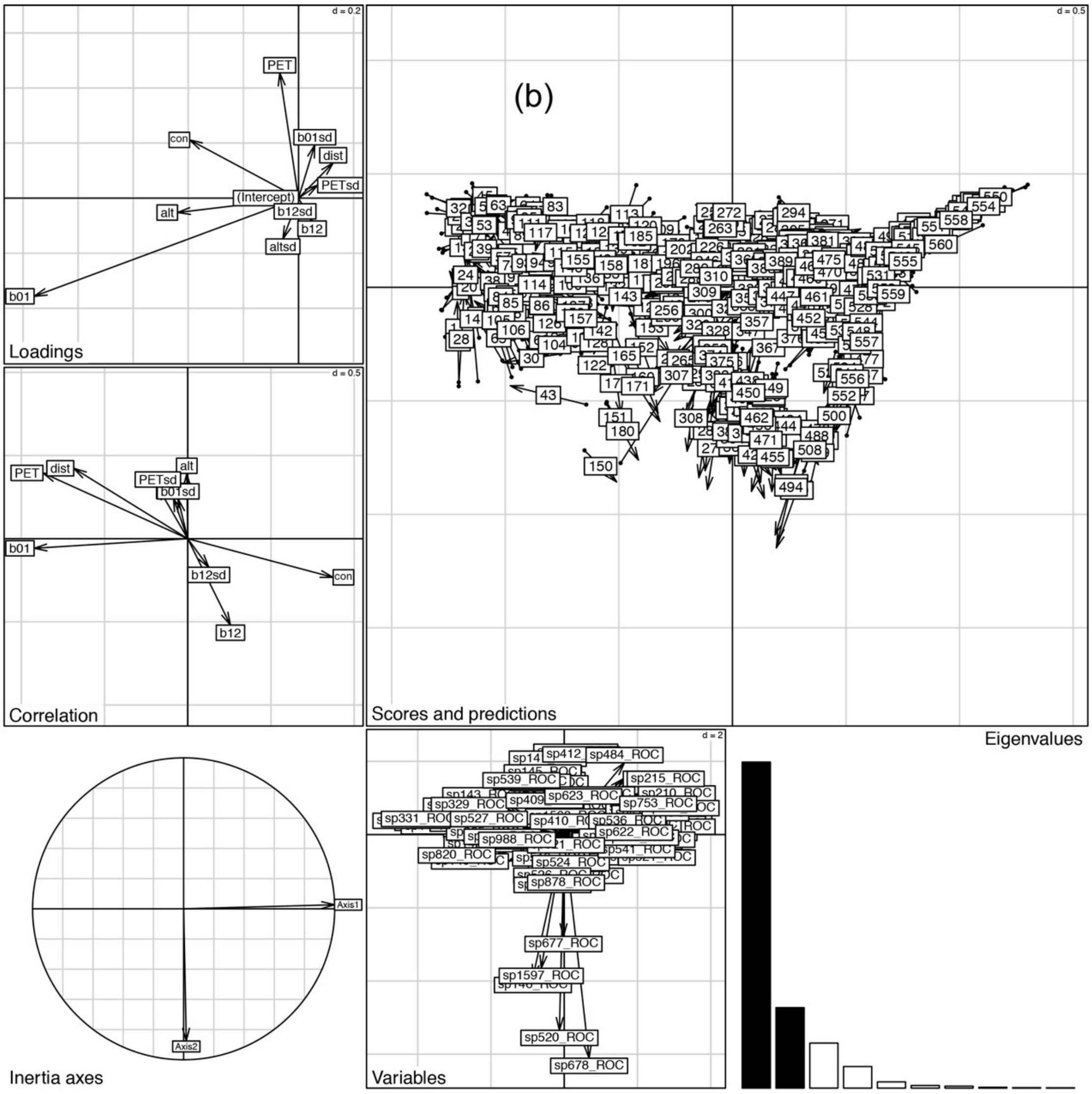

Figure S6c. Results of canonical correspondence analysis of species composition of mosses and environmental variables (see Table 1).

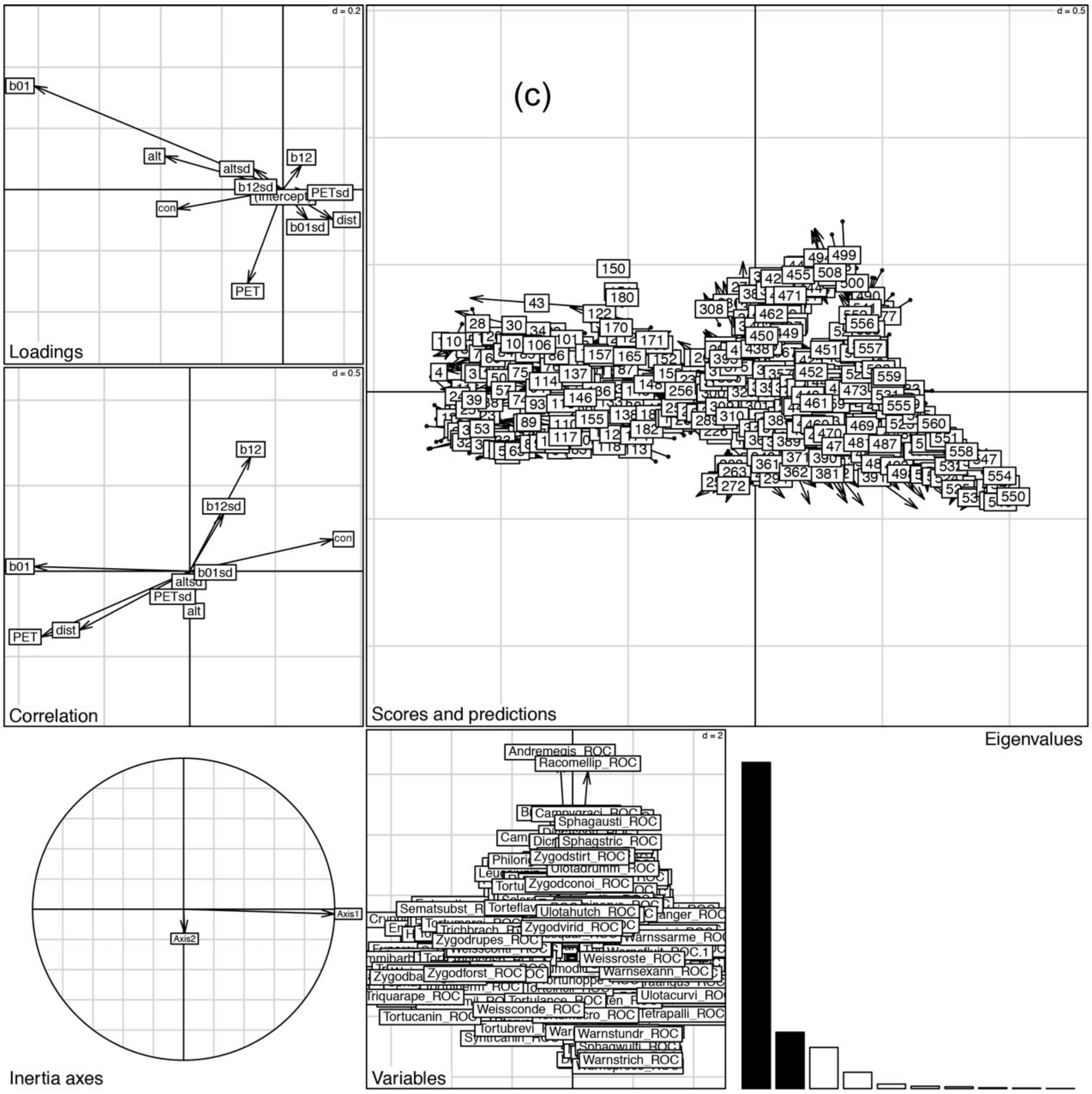

Figure S6d. Results of canonical correspondence analysis of species composition of liverworts and environmental variables (see Table 1).

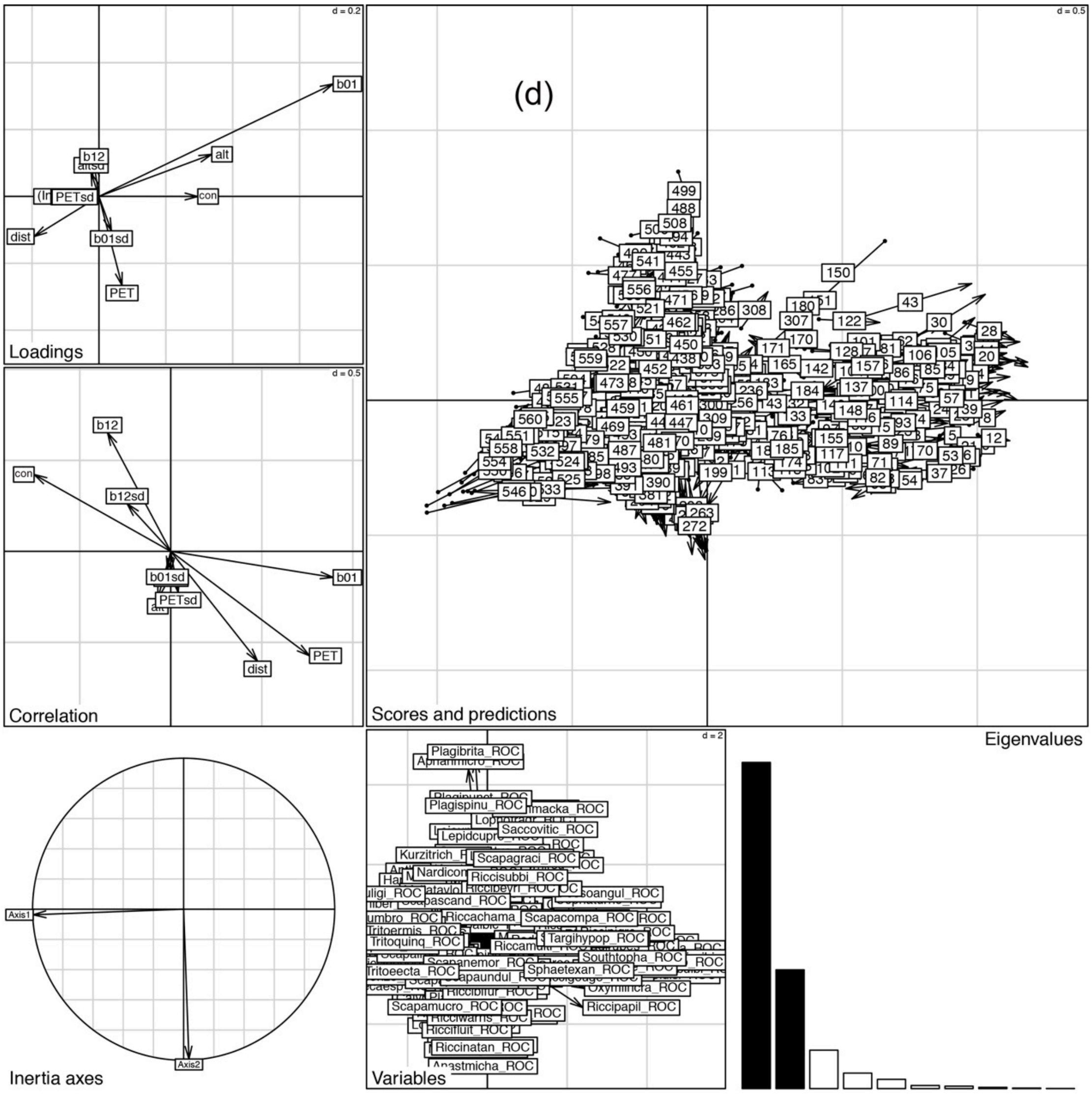

Figure S7. Residuals of the Procrustes analysis based in the comparison of two CCA. Map generated by R.G. Mateo using the ArcMap extension in ArcGIS 10.2 (ESRI Inc., Redlands, CA, USA, http://www.esri.com). (a) CCA of species composition of mosses and environmental variables vs. CCA of species composition of spermatophytes and environmental variables**.** (b) CCA of species composition of liverworts and environmental variables vs. CCA of species composition of spermatophytes and environmental variables. (c) CCA of species composition of ferns and environmental variables vs. CCA of species composition of spermatophytes and environmental variables

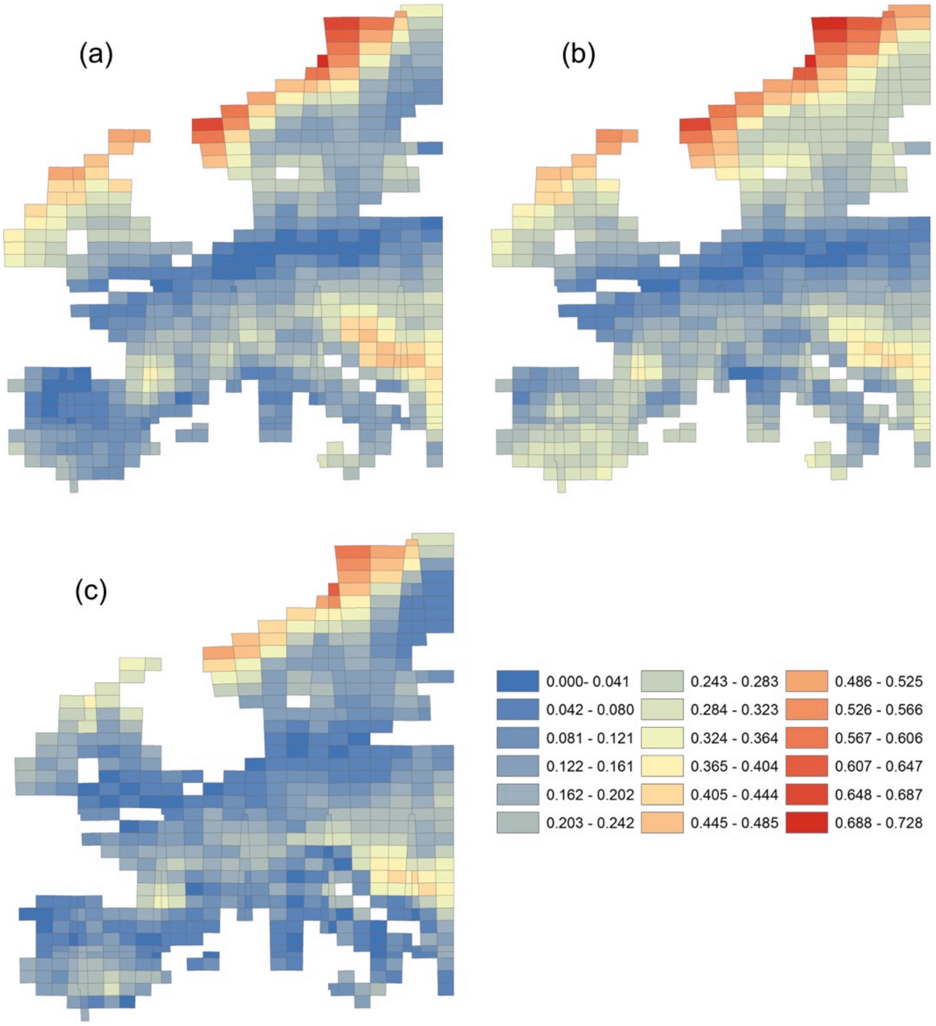

References

1. Lee, S.I. Developing a bivariate spatial association measure: an integration of Pearson's r and Moran's I. *J. Geogr. Syst.* **3**, 369-385 (2001).

2. Bivand, R.S., Gómez-Rubio, V. & Rue, H. Spatial data analysis with R-INLA with some extensions. *J. Stat. Softw.* **63**, 31 (2015).

3. Goodchild, M.F. *Spatial Autocorrelation*. (Geo Books, Norwich, UK, 1986).

4. Oksanen, J. *Multivariate analysis of ecological communities in R: vegan tutorial* [online]. Available from http://cc.oulu.fi/~jarioksa/opetus/metodi/vegantutor.pdf [accessed May 2015].

5. Dray, S., Pélissier, R., Couteron, P., Fortin, M.J., Legendre, P., Peres-Neto, P.R., Bellier, E., Bivand, R., Blanchet, F.G., De Cáceres, M., Dufour, A.B., Heegaard, E., Jombart, T., Munoz, F., Oksanen, J., Thioulouse, J. & Wagner, H.H. Community ecology in the age of multivariate multiscale spatial analysis. *Ecol. Mon*. **82**, 257-275 (2012).

6. Dray, S., Dufour, A.B. & Chessel, D. The ade4 package-II: Two-table and K-table methods. *R News* **7**, 47-52 (2007).

Supplementary Methods 5: Comparison of the latitudinal patterns of species richness for mosses, liverworts, ferns and spermatophytes

To illustrate the results obtained in the pixel analyses we provide the following graphs of the latitudinal patterns of species richness (SR) of mosses, liverworts, ferns and spermatophytes (Fig. S8a), as well as some environmental variables and other data used in the study calculated for latitudinal bands of 100 km stretch across Europe (Fig. S8b-i).

In order to represent adequately the latitudinal gradients of SR, the observed SR values *(OS_i*; in our case, modelled through stacked species distribution models, S-SDMs) for every latitudinal band *i* of 100 km stretch were calculated for every taxonomic group. Each latitudinal band has an effective area A_i (the sum of the areas of the pixels included in the band, excluding sea surfaces). In the measure that SR is dependent upon area, we need an estimation of the expected SR values *(ES_i)* if SR was an exact function of area. The most consistent mathematical relationship between SR *(S)* and area *(A)* (species-area relationship, SAR) is the power function[1]: $S=cA^z$. For deriving a value of *c*, we assume that total species presences are the same in expected and observed SR sets, i.e. $\Sigma ES_i=\Sigma OS_i$. Hence, $ES_i=A_i^{\,z}\ \Sigma OS_i\,/\,\Sigma(A_i^{\,z})$.

For the estimation of the value of *z*, SR was calculated for groups of contiguous pixels ranging in area from ~10^4 km^2 (1 pixel) to ~$64*10^4$ km^2 (64 pixels), completely nested within them and covering almost all Europe. Pixels covering sea surfaces were excluded from this analysis. The species-area relationship was calculated via regression of SR on area (Dengler 2009). The *z* values obtained were slightly higher for spermatophytes (0.185) than for liverworts (0.165), mosses (0.163) and ferns (0.133). These values are common for species-area curves from large mainland areas[2]. According to these SAR equations for Europe, the species richness corrected for area

effects *(CSR_i)* were calculated as: $CSR_i = OSR_i - ESR_i + MSR$, being *MSR* the mean species richness across the latitudinal gradient.

A simple test to prove the existence of a latitudinal gradient in SR across latitudinal bands independent of their area was carried out. Linear regressions of the SR values normalized by area on latitude were calculated and we checked if their slopes (a) differ significantly from zero. A significant slope was found for mosses and spermatophytes, positive for the former ($a=6.87\pm1.692$, $t_{35}=4.06$, $p=.0003$) and negative for the latter ($a=-24.26\pm2.251$, $t_{35}=-10.78$, $p<10^{-11}$). Slope was non-significant for ferns ($a=0.063\pm0.159$, $t_{35}=.396$, $p=0.69$) and only marginally significant for liverworts ($a=-1.77\pm0.872$, $t_{35}=-2.03$, $p=0.0501$).

Figure S8: Graphs of the latitudinal patterns of species richness (SR) of mosses, liverworts, ferns and spermatophytes, as well as some environmental variables and other data used in the study calculated for latitudinal bands of 100 km stretch across Europe.

Figure S8a: Predicted numbers of species of spermatophytes (green), ferns (black), mosses (red) and liverworts (blue) in 100 km latitudinal bands across Europe. Dashed lines indicate crude SR values predicted by S-SDMs, solid lines correspond to SR values normalized according to species-area relationships. In the lower graph SR values of the four groups have been rescaled to allow comparisons.

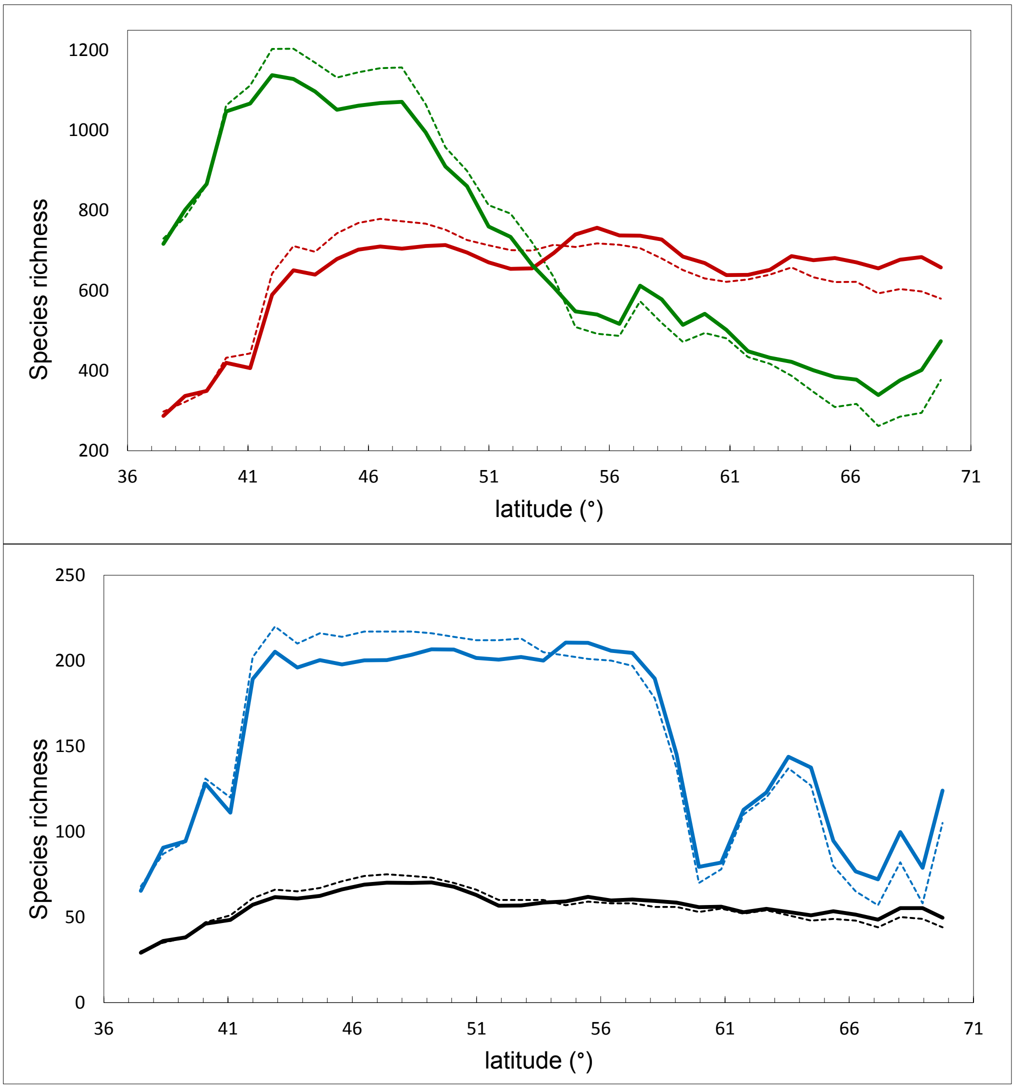

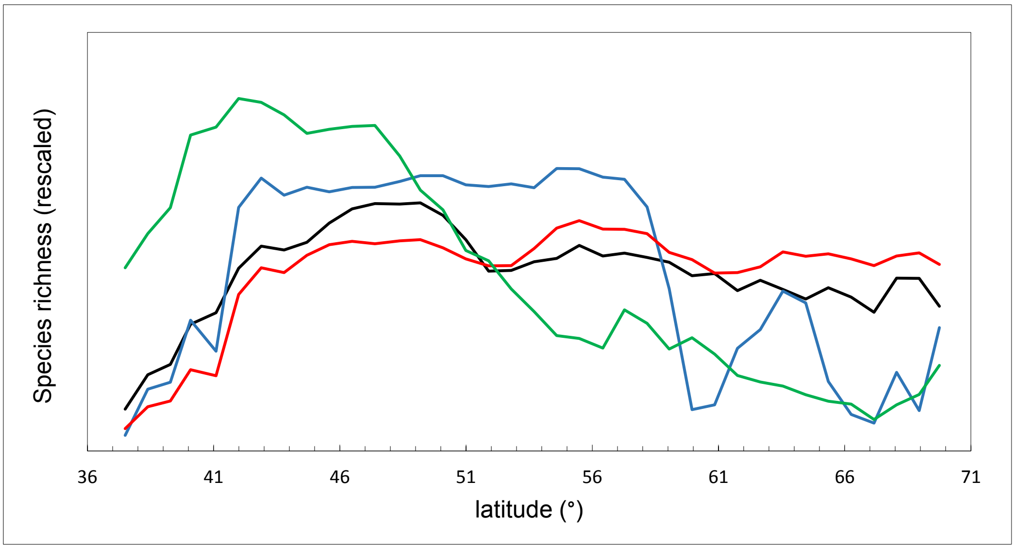

Species richness (rescaled)
36
41
46
51
56
61
66
71
latitude (°)

Figure S8b: Mean and SD elevation range by latitudinal band.

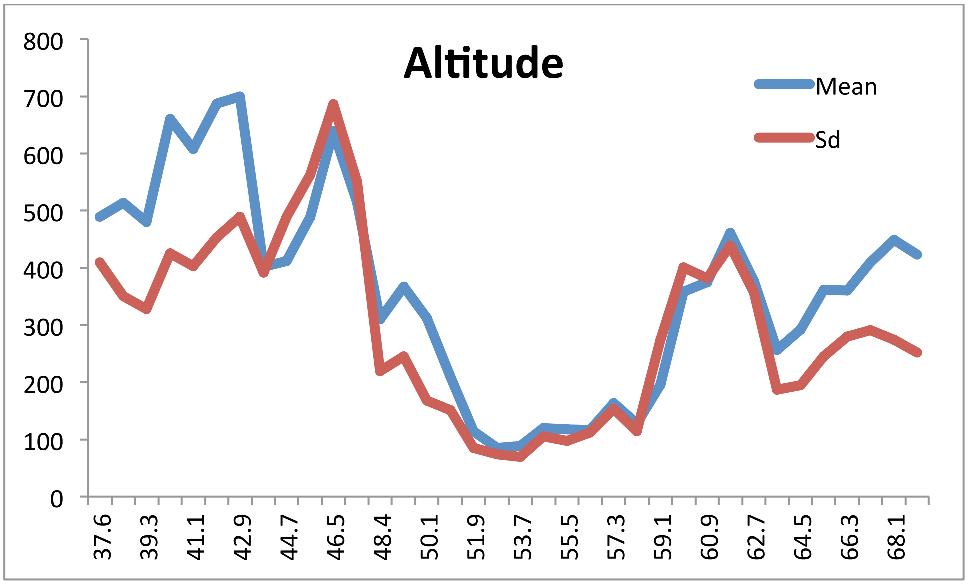

Figure S8c: Mean and SD annual temperature (ºC x 10) by latitudinal band.

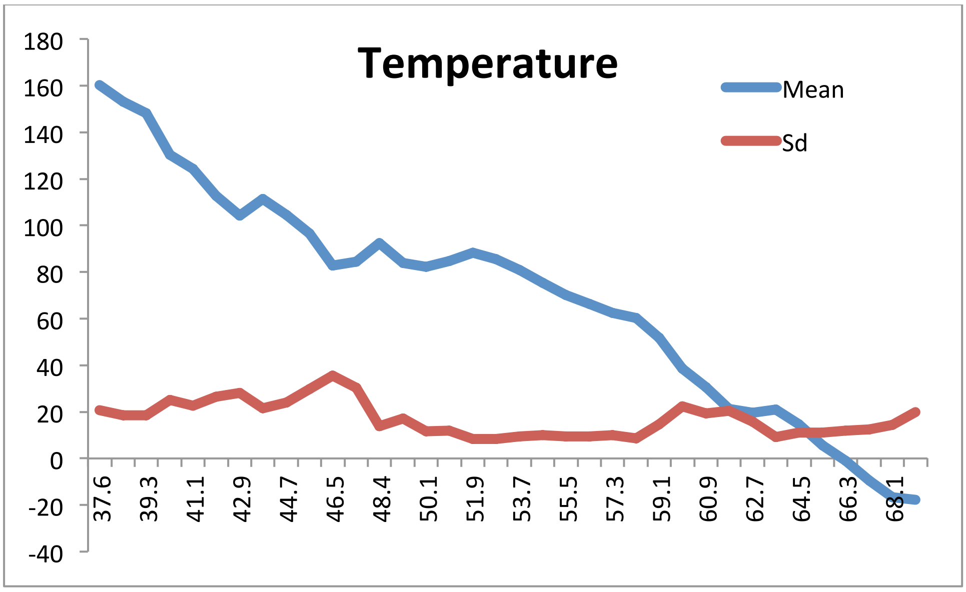

Figure S8d: Mean and SD annual precipitation (mm/m^2) by latitudinal band.

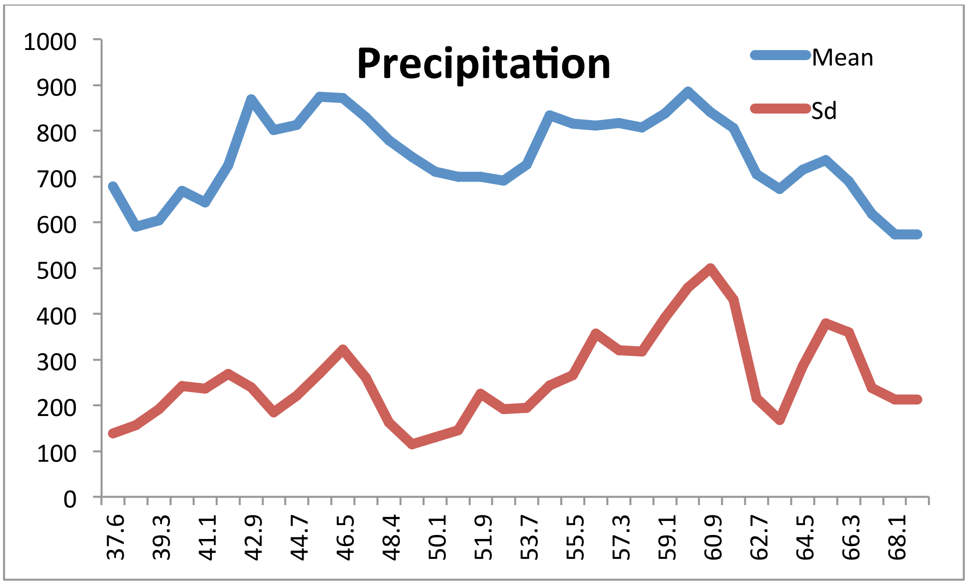

Figure S8e: Mean and SD annual PET (mm m^{-2} day^{-1}) by latitudinal band.

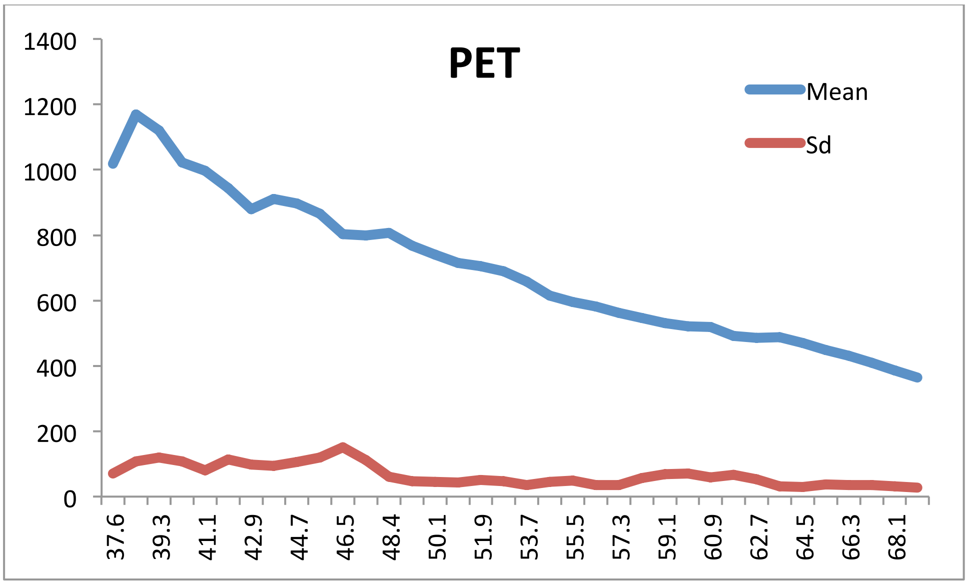

Figure S8f: Area covered by this study by latitudinal band.

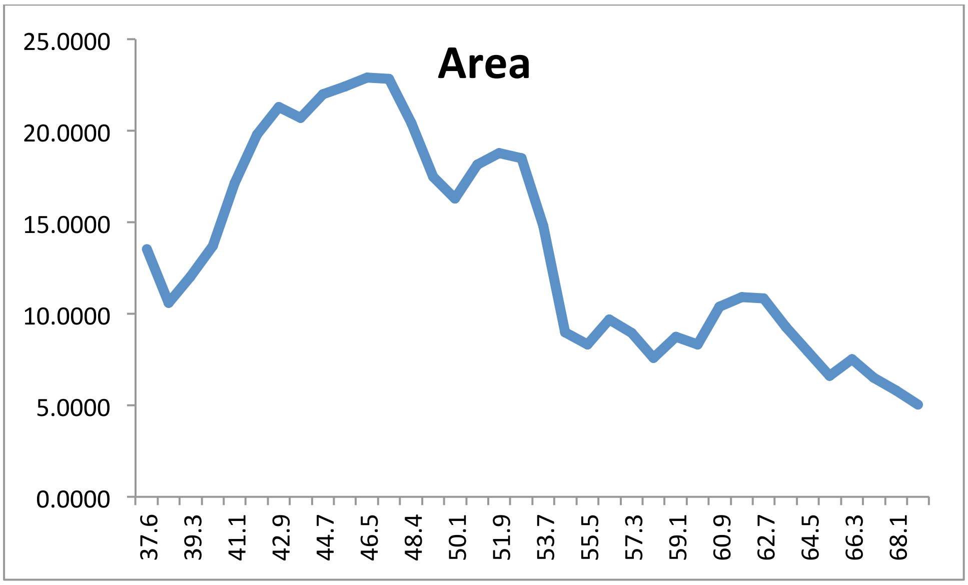

Figure S8g: Number of collection for bryophytes in the database employed in this study, by latitudinal band.

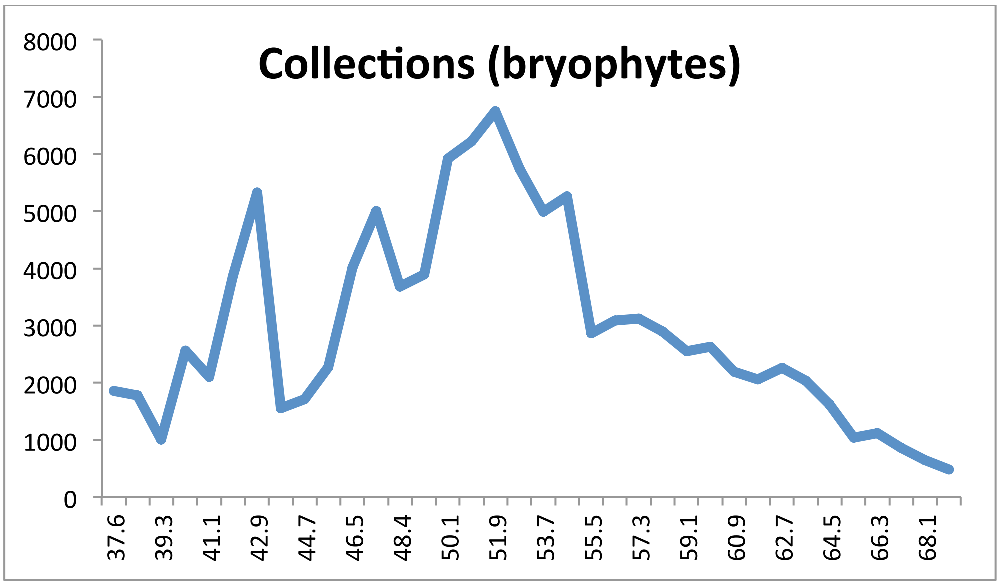

Figure S8h: Number of collection for bryophytes in the database employed in this study normalized by area, by latitudinal band.

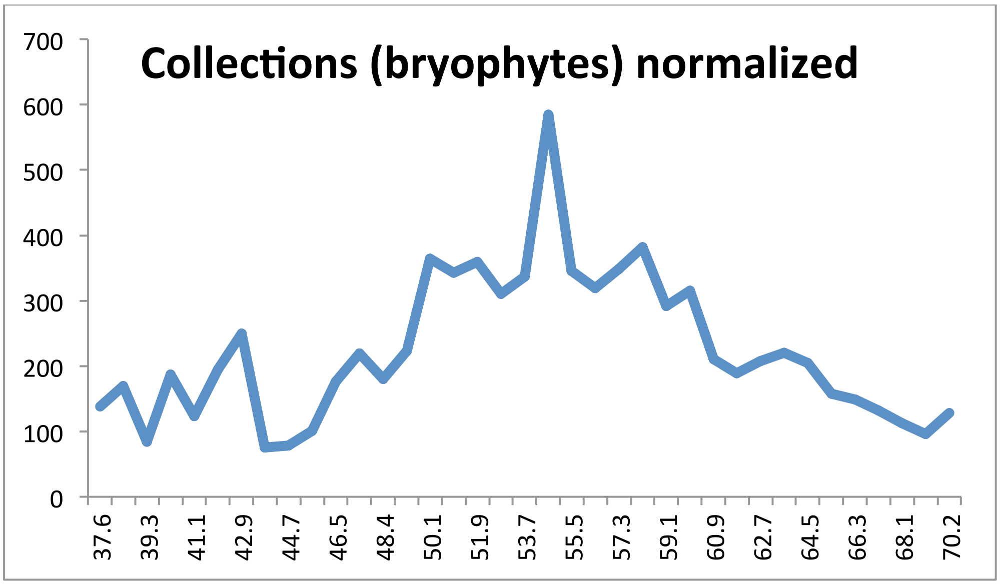

Figure S8i: Number of collection for bryophytes in the GBIF database by latitudinal band.

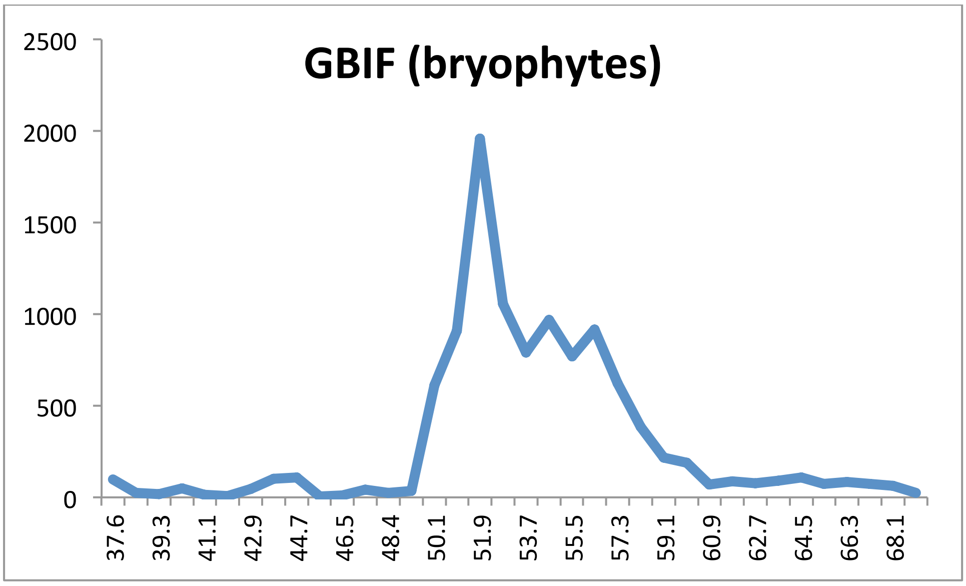

References

1. Dengler J. Which function describes the species-area relationship best? A review and empirical evaluation. *J. Biogeogr.* **36**, 728-744 (2009).

2. Rosenzweig M.L. *Species diversity in space and time*. (Cambridge University Press, Cambridge, 1995).
